# Machine Learning Reveals Quantitative Amino Acid Preferences in *Bifidobacterium longum* Growth

**DOI:** 10.1111/1751-7915.70367

**Published:** 2026-05-05

**Authors:** Hiroki Kaneko, Kana Kadowaki, Shin Yoshimoto, Toshitaka Odamaki, Bei ‐ Wen Ying

**Affiliations:** ^1^ Biotics Research Institute Morinaga Milk Industry co., Ltd. Zama Kanagawa Japan; ^2^ School of Life and Environmental Sciences University of Tsukuba Tsukuba Ibaraki Japan; ^3^ MiCS University of Tsukuba Tsukuba Ibaraki Japan

**Keywords:** amino acid, *Bifidobacterium*, culture medium, machine learning

## Abstract

*Bifidobacterium longum* is a prevalent human gut symbiont whose carbohydrate metabolism is well characterized, whereas the quantitative contribution of amino acids to growth remains unclear. Here, we combined genome‐based pathway analysis, growth phenotyping in chemically defined media, and iterative machine‐learning‐guided medium design to quantify amino acid preferences in *B. longum* subsp. *longum* JCM 1217^T^. Genome analysis predicted cysteine as the sole auxotrophy, and experiments confirmed that cysteine alone supported growth but did not restore the high maximum cell density and short lag time achieved with a complete amino acid mixture. Regression models and genetic algorithms identified amino acid formulations in the selected optimized compositions that reduced total amino acid input by 66.5% under glucose and 77.2% under lactose while maintaining growth comparable to complete medium. SHAP analysis highlighted tyrosine as the main determinant of maximum cell density, whereas glutamate, leucine, and valine consistently shortened lag time. These results show that amino acid requirements in *B. longum* extend beyond binary auxotrophy and provide a machine‐learning framework for designing streamlined defined media.

## Graphical abstract

Machine‐learning–guided optimization of amino acid composition revealed amino acid preferences in *Bifidobacterium longum* beyond auxotrophy. Cysteine was essential for growth, whereas under reduced amino acid supply, tyrosine predominantly influenced growth yield, and glutamate, leucine, and valine were key determinants of lag time.

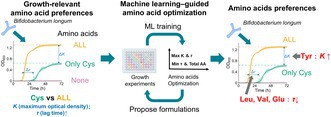

## 1 Introduction

*Bifidobacterium* spp. are dominant members of the infant gut microbiota and remain detectable in many adults (Kato et al. 2017). Several strains are used as probiotics and have been reported to improve bowel‐movement related outcomes and modulation of immune responses (Henrick et al. 2021; Takeda et al. 2023). Research on *Bifidobacterium* has largely focused on carbohydrate metabolism—particularly the utilization of human milk oligosaccharides (HMOs), host‐derived mucins, and plant polysaccharides—and on the physiological roles of production of short‐chain fatty acids (Sakanaka et al. 2019; Katoh et al. 2023; Arzamasov et al. 2025). Recently, the physiological relevance of amino acid metabolism has gained attention. Infant‐associated bifidobacteria convert aromatic amino acids (Trp/Phe/Tyr) into aromatic lactic acids, including indole‐3‐lactic acid (ILA), phenyllactic acid (PLA), and 4‐hydroxyphenyllactic acid (4‐OH‐PLA), which modulate host immunity—for example through AhR/HCAR3 signalling or suppression of IgE production (Laursen et al. 2021; Myers et al. 2026).

Amino acids also represent a primary source of organic nitrogen for microbial growth; thus, both the quantity and form of exogenous amino acids can influence growth phenotypes such as the maximum cell density (K), specific growth rate (r), and lag time (τ). Chemically defined media (CDM) have clarified amino acid auxotrophy in *Bifidobacterium*, but most studies rely on binary assessments of “growth” versus “no growth” (Ferrario et al. 2015). In practice, many amino acids exert quantitative and dose‐dependent effects even when they are not strictly required for growth. For instance, isoleucine and tyrosine were identified as growth‐promoting amino acids in *B. longum* 105‐A despite the absence of strict auxotrophy (Sakaguchi et al. 2013). These graded effects, however, have not been systematically evaluated in a quantitative and multivariate manner across the amino acid landscape.

Similarly, Genome‐scale metabolic models (GEMs) are useful for predicting pathway completeness and potential auxotrophies (Starke et al. 2023). However, their ability to predict quantitative growth improvements from individual amino acids is limited due to uncertainties introduced by homology‐based reconstruction, gap filling (Chen et al. 2023), and the absence of regulatory information (e. g, gene expression and transport regulation). In this context, we define “amino acid preference” as the extent to which exogenous amino acids quantitatively improve r, τ, or K, independent of strict growth requirement. Under amino‐acid–rich conditions, these preferences are largely masked, whereas restricting total amino‐acid supply can reveal differences in biosynthetic costs, cellular demands, and uptake efficiencies. Moreover, the combinatorial space of amino acid compositions renders exhaustive experimental testing infeasible, necessitating data‐driven approaches that can capture nonlinear and interactive effects.

To quantitatively evaluate amino acid preferences, we employed an integrated framework combining genome‐based pathway analysis, high‐throughput growth phenotyping in chemically defined media, machine‐learning regression, and multi‐objective optimization in an iterative propose–test–update cycle. Using this approach in *Bifidobacterium longum* subsp. *longum* JCM 1217^T^, we aimed to identify amino acid compositions that support robust growth while minimizing total amino acid input, thereby revealing preferences that extend beyond classical auxotrophy.

## 2 Experimental Procedures

### 2.1 KEGG Annotation

The complete genome sequence of *B. longum* JCM 1217^T^ (GenBank accession: AP010888) was retrieved from NCBI. Gene prediction and functional annotation were performed using Bakta v1.9.3 with bakta_db v5.0 (full) under default parameters (Schwengers et al. 2021). Protein sequences from the Bakta output were used for KEGG Ortholog (KO) assignment with KOfamScan v1.3.0 (Aramaki et al. 2020) and eggNOG‐mapper v2.1.13 (Cantalapiedra et al. 2021).

For KOfamScan, only hits meeting the recommended adaptive score threshold were retained. The KOfam HMM profiles used were from the November 2025 release. For eggNOG‐mapper v2, MMseqs2 v18‐8cc5c (Steinegger and Söding 2017) was used with the “more sensitive” setting, and databases were downloaded in November 2025 using download_eggnog_data.py.

KEGG Ortholog (KO) annotations from the two methods were integrated by prioritizing KOfamScan. KOfamScan‐derived KOs were retained, and eggNOG‐mapper KOs were added only when no KOfamScan KO was assigned for a gene. When multiple KOs were assigned to a gene, all were retained. The integrated KO table is provided as Table S1.

### 2.2 Definition and Completeness Assessment of Amino Acid Biosynthetic Pathways for *Bifidobacterium*

Biosynthetic pathways for 20 amino acids (21 pathway entries, treating cysteine as two alternatives) were curated as custom JSON pathway definitions based on KEGG MODULE definitions implemented in KEMET v1.0.0 (Palù et al. 2022). In total, 72 enzymatic steps were evaluated (Figures S1–S7).

A step was marked present if any corresponding KO (including alternatives) was detected in the integrated KO set; otherwise, it was marked missing. Completeness was computed as the percentage of present steps, and pathways were classified as “ALL enzymes annotated” (100%) or “Missing enzyme(s)” (< 100%). All analyses were implemented in a reproducible Python/R pipeline (bifidobacterium‐aa‐biosynthesis).

### 2.3 Preparation of Concentrated Stocks for CDM With Variable Amino Acid Concentrations

A chemically defined medium (CDM) was prepared following Schöpping et al., using a base formulation lacking carbohydrates and amino acids (Schöpping et al. 2021). A 2 × base medium (Table S2) was prepared in Milli‐Q water, adjusted to pH 6.5, sterilized by 0.22‐μm PVDF filtration, aliquoted and stored at −30°C.

Glucose or lactose was added from 100 g L^−1^ sterile stocks to obtain 10 g L^−1^ in the 1 × medium. Glucose was used as a standard reference carbon source widely employed in conventional cultivation media. Lactose was selected as a milk‐associated disaccharide, as it is the major carbohydrate in human breast milk and is relevant to the infant gut environment where bifidobacteria are abundant. The use of these two carbon sources allowed us to assess whether amino acid preferences are carbon‐source dependent.

L‐amino acids were prepared as individual 20 mM sterile stocks and stored at −30°C. Due to solubility constraints, tyrosine (Tyr) stock solutions could not be prepared at 20 mM; therefore, multiple 2× base‐medium variants differing only in Tyr concentration were used.

The 1× medium (5 mL total volume) was prepared by mixing 2.5 mL of 2 × base medium, 500 μL sugar stock, amino acid stock volumes corresponding to target concentrations, and sterile water to volume. Amino acid stocks were added either undiluted or after 10‐ or 100‐fold dilution, depending on the target medium composition, such that 100 μL achieved the intended final concentration. Prepared media were pre‐reduced overnight in an anaerobic chamber.

### 2.4 Anaerobic Conditions

All medium preparation, inoculation, cultivation, and cell density measurements were performed in an anaerobic chamber (Bactron 600, Sheldon Manufacturing) maintained at N_2_:H_2_:CO_2_ = 90:5:5 (v/v/v). Cell density was monitored as OD600 (absorbance reading at 600 nm), which primarily reflects light scattering by the culture and is used here as a proxy for cell density. Cultures were incubated at 37°C in an incubator inside the chamber.

### 2.5 Preparation of Strain Stocks

*Bifidobacterium. longum* JCM 1217^T^ was obtained from the Japan Collection of Microorganisms (JCM). For the primary stock, cultures were grown anaerobically in MRS medium with 0.05% (w/v) L‐cysteine at 37°C for 16 h, and passaged twice (3% inoculation, 16 h each). After the second passage, 50% (v/v) glycerol was added at a 4:1 (culture: glycerol) ratio to obtain 10% final glycerol. Aliquots were stored at −80°C.

For the working stock, the primary stock was revived in CDM with L‐cysteine as the sole amino acid (CDM‐Cys) and adapted by two passages (3% (v/v) inoculation, 24 h each). Exponential‐phase cultures (OD600 = 0.4 ± 0.1) were mixed with anaerobic 50% (v/v) glycerol at 4:1 (culture: glycerol) to obtain 10% glycerol and stored at −80°C. Each aliquot was used once.

### 2.6 Growth Measurement in 96‐Well Plates

To reduce condensation artefacts, plate lids were rendered hydrophilic following Brewster using 0.05% Triton X‐100/20% ethanol and dried at 37°C in the anaerobic chamber (Brewster 2003).

Working stocks were thawed and diluted sequentially: −80 μL stock + 720 μL medium (10‐fold dilution), −110 μL of this dilution +990 μL medium (100‐fold dilution), yielding a final inoculation rate of 1% (v/v).

Cultures (200 μL per well) were dispensed into inner wells of a 96‐well microplate (Corning, REF 353072) with four or five replicate wells per condition. Peripheral wells contained sterile water. Plates were sealed with Parafilm and measured in an Epoch 2 plate reader (Agilent BioTek) at 37°C for 72 h (1‐h intervals, 73 reads). Before each measurement, plates were shaken to resuspend cells. OD600 was measured using endpoint reads (8 measurements per time point).

Following cultivation, cultures were centrifuged (8000 g, 10 min) and supernatants stored at −30°C.

### 2.7 Estimation of Growth‐Curve Parameters (K, r, τ)

Growth‐curve analysis was performed in Python using a unified pipeline. OD values were baseline‐corrected by subtracting the initial OD. The maximum cell density (K) was defined as the mean of the three points surrounding the maximum value. Conditions with K < 0.1 were classified as no growth; τ was set to NaN and r was set to 0.

Lag time (τ) was defined as the first time point at which baseline‐corrected OD exceeded 0.02 and increased monotonically over five subsequent points. Exponential‐phase onset was identified using the PELT algorithm (L2 model) in ruptures v1.1.10. Penalty parameters were increased iteratively until regression of log(OD) achieved R^2^ ≥ 0.999 (max 10 iterations). The exponential phase extended until OD reached 60% of K. The specific growth rate (r) was estimated by linear regression of log (OD) using scikit‐learn LinearRegression. Parameter means and SD were computed from replicate wells.

### 2.8 Model‐Guided Optimization of Amino Acid Composition

To enable quantitative medium design, we used amino acid compositions as inputs and growth parameters—maximum cell density (K), specific growth rate (r), and lag time (τ)—as outputs. Regression models were built using CultureSage v1.0.0 to predict K, r, and τ from amino acid compositions. Input features were natural‐log–transformed amino acid concentrations ln(concentration + ε) with ε = 10^−9^.

Eight regression algorithms were compared: XGBoost v3.0.5, LightGBM v4.6.0, CatBoost v1.2.8, RandomForest (scikit‐learn v1.7.1), Support Vector Regression (SVR; RBF kernel), Ridge regression, Multilayer perceptron (MLP), and k‐nearest neighbours (KNN). Hyperparameters were optimized using Bayesian optimization (scikit‐optimize gp_minimize) with Expected Improvement. Iteration budgets were assigned using model‐complexity and data‐size multiplier (range 10–300 iterations). Model performance was evaluated via bootstrap leave‐one‐out cross‐validation (LOOCV) with 200 resamples; evaluation metrics included R^2^, RMSE, and MAE (Table S5). For each target variable in each round, the algorithm with the lowest bootstrap‐LOOCV RMSE was selected as the surrogate model for NSGA‐II optimization; the selected algorithm therefore varied across rounds, targets, and carbon sources (Table S5). In the final round (Round 6), which incorporated all accumulated data, CatBoost (K) and RandomForest (r and τ) were selected under glucose, and XGBoost was selected for all three targets under lactose. LOOCV was chosen because each iteration trains on N‐1 samples, maximizing data use when the number of conditions per round is small (58–148).

Using the trained models as surrogate predictors of K, r, and τ, we next searched for amino acid compositions that balance growth performance and minimal amino acid input. Amino acid compositions were optimized using NSGA‐II (Non‐dominated Sorting Genetic Algorithm II, DEAP v1.4.3) with four objectives: maximize K, maximize r, minimize τ, and minimize total amino acid input. A two‐stage knee‐point selection was implemented to select 15 conditions from the Pareto front: (1) 30 candidates selected using distance, curvature, and dominance metrics; (2) percentile thresholding and max–min diversity filtering.

Iterative, model‐guided optimization was performed in a propose–test–update cycle. Based on the confirmed cysteine auxotrophy (Section 3.1), cysteine was fixed and excluded from the optimization variables in all rounds. In Round 0 (R0), media were designed by varying each amino acid across four levels (0, 4, 40, and 400 μM) plus a complete reference, yielding 58 conditions per carbon source. Models trained on R0 were used to propose 15 candidates for the next round. This cycle was repeated for R1–R6, resulting in 148 total conditions per carbon source.

Finally, model interpretation was performed to identify which amino acids most strongly influenced predictions. Feature contributions were quantified using SHAP v0.48.0. TreeExplainer was used for tree‐based models and KernelExplainer for others. SHAP uncertainty was estimated from 200 bootstrap resamples using a background set of 100 randomly selected samples.

### 2.9 Data Visualization

Figures were generated in R v4.5.1 using ggplot2 v4.0.1, with patchwork v1.3.2 and cowplot v1.2.0 for layout, and pheatmap v1.0.13 for heatmaps. SHAP values were computed in Python (Python v3.12.7; shap v0.48.0) and imported into R for visualization. Colours were based on the Okabe–Ito palette.

### 2.10 Statistics

Statistical analyses were performed in Python 3.12.7 (SciPy v1.16.3) and R v4.5.1. Two‐group comparisons used two‐sided Welch's *t*‐test (α = 0.05), and multi‐group comparisons used one‐way ANOVA followed by Tukey's HSD. Where shown, significance is indicated as *p < 0.05, **p < 0.01, and ***p < 0.001.

## 3 Results

### 3.1 Genomic Prediction and Experimental Validation of Amino Acid Auxotrophy

**FIGURE 1:**
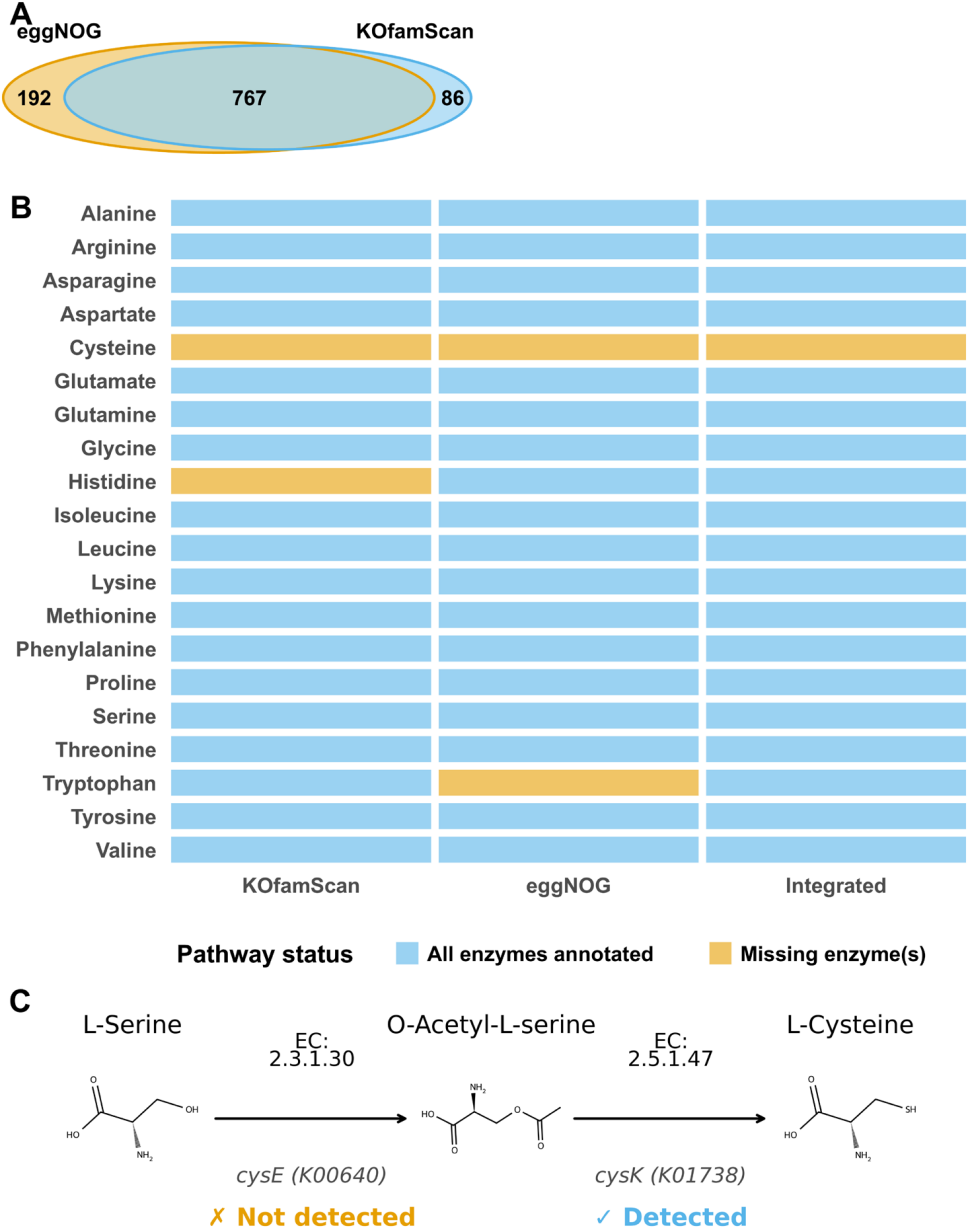
Integration of KEGG Ortholog (KO) annotations from KOfamScan and eggNOG‐mapper (A) Venn diagram showing overlap and unique KO assignments between KOfamScan (853 KOs), eggNOG‐mapper (959 KOs), and the integrated KO set (1045 unique KOs). (B) Comparison of amino‐acid biosynthetic pathway completeness as inferred from KOfamScan, eggNOG‐mapper, and the integrated KO set. “ALL enzymes annotated” indicates that all enzyme‐encoding genes (KOs) required for de novo biosynthesis of a given amino acid were detected in the genome, whereas “Missing enzyme(s)” indicates that one or more essential biosynthetic enzymes were missing. Based on the integrated KO analysis, all pathways were classified as complete except for cysteine. (C) Cysteine biosynthesis pathway showing the sequential conversion from L‐serine to O‐acetyl‐L‐serine to L‐cysteine with chemical structures. *cysK* (K01738; EC 2.5.1.47, cysteine synthase) was detected in the genome, whereas *cysE* (K00640; EC 2.3.1.30, serine O‐acetyltransferase) was not detected.

As a prerequisite for experimental design and optimization, we first assessed the amino acid biosynthetic capabilities of *B. longum* JCM 1217^T^. To predict amino acid biosynthetic capabilities of *B. longum* JCM 1217^T^, we annotated the complete genome using KOfamScan and eggNOG‐mapper and integrated the resulting KEGG Ortholog (KO) assignments (Figure 1; Table S1). We evaluated the 20 amino acid biosynthetic modules defined for *Bifidobacterium*, comprising 72 enzymatic steps in total. Genes corresponding to 71 of the 72 steps (98.6%) were detected, yielding complete biosynthetic pathways for 19 amino acids (Table S3). In contrast, the cysteine biosynthesis module lacked.

In contrast, the cysteine biosynthesis module was incomplete: the gene encoding serine O‐acetyltransferase (*cysE*; K00640), which catalyzes the conversion of serine to O‐acetylserine, was not detected, whereas cysteine synthase (*cysK*; K01738) was present. This indicates a likely cysteine‐auxotrophy (Figure 1).

**FIGURE 2:**
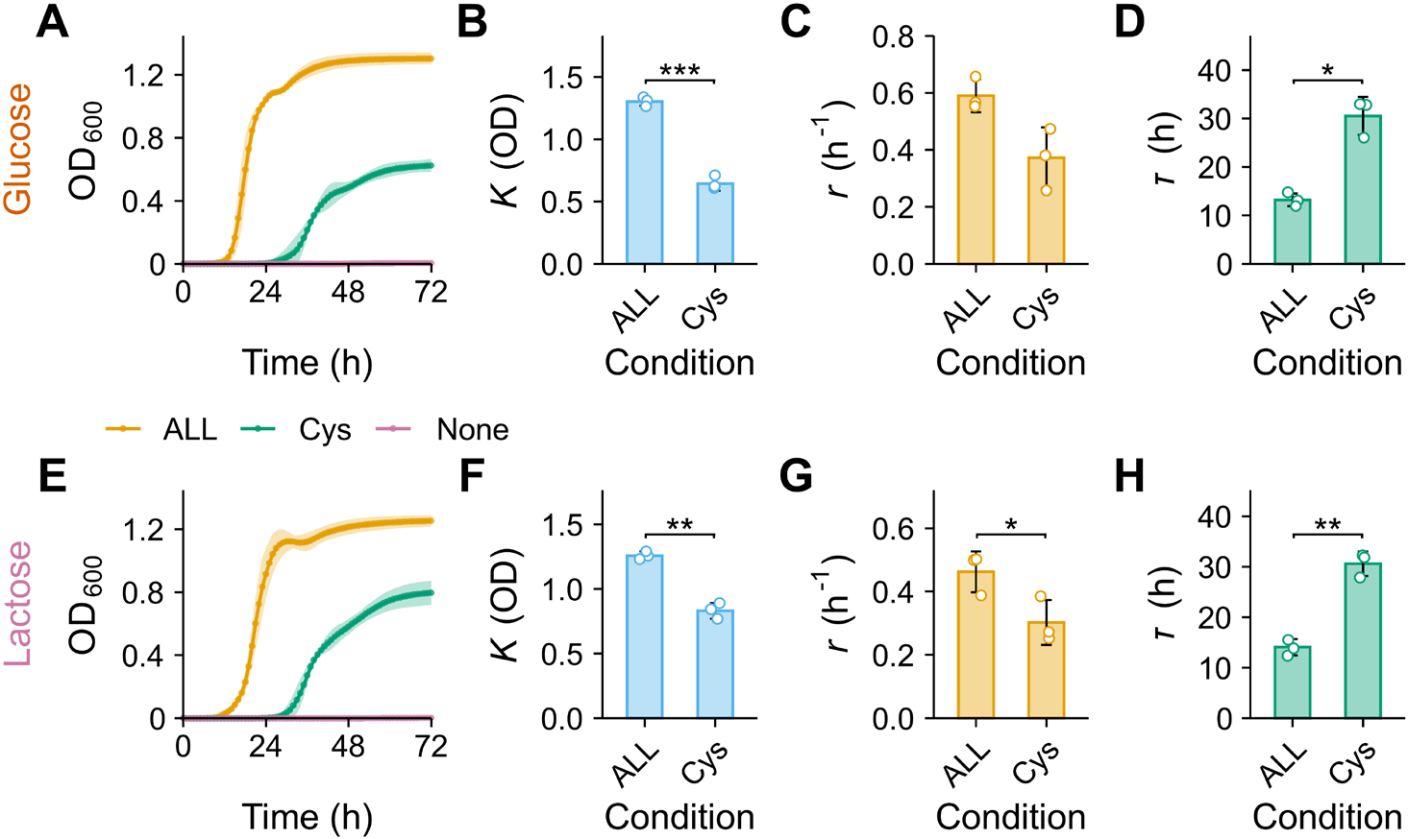
Experimental validation of cysteine auxotrophy in *B. longum* JCM 1217^T^ (A, E) Growth curves over 72 h under three amino acid conditions: ALL, all 20 amino acids at 400 μM each (orange); Cys, cysteine only at 400 μM (green); None, no amino acids (pink). Growth was measured with glucose (A) or lactose (E) as the carbon source. Lines represent mean OD600 ± SD (*n* = 3 independent experiments; 4, 5 technical replicates each). No growth was observed in None. (B–D, F–H) Growth parameters comparing ALL and Cys under glucose (B–D) and lactose (F–H): Maximum cell density (K) (B, F), specific growth rate r (C, G), and lag time (τ) (D, H). Bars show mean ± SD (*n* = 3 independent experiments; 4–5 technical replicates each); points indicate replicates. Welch's *t*‐test: *p < 0.05, **p < 0.01, ***p < 0.001.

We next experimentally validated this genomic prediction. Using glucose or lactose as the carbon source, we measured 72‐h growth under three amino acid conditions: (i) ALL (all 20 amino acids at 400 μM), (ii) Cys (cysteine only at 400 μM), and (iii) None (no amino acids) (Figure 2A,E). Robust growth was observed in the ALL condition, yielding τ = 13.20 ± 1.31 h, *r* = 0.590 ± 0.058/h, and K = 1.304 ± 0.036 (glucose), and τ = 14.07 ± 1.62 h, *r* = 0.462 ± 0.065/h, and K = 1.258 ± 0.034 (lactose). In contrast, no detectable growth occurred under the None condition for either carbon source (Figure 2A,E). Notably, the Cys condition supported growth, but resulted in significantly lower K and significantly prolonged τ compared with the ALL condition under both carbon sources (Figure 2B–D,F–H). Thus, exogenous cysteine alone restored basic growth capability but failed to reproduce the maximal growth performance achieved with a full amino acid supply. These findings demonstrate that binary auxotrophy predictions based on pathway completeness do not fully capture the observed differences in growth phenotypes under varying amino acid conditions.

### 3.2 Construction of Machine‐Learning Models and Iterative, Model‐Guided Optimization

**FIGURE 3:**
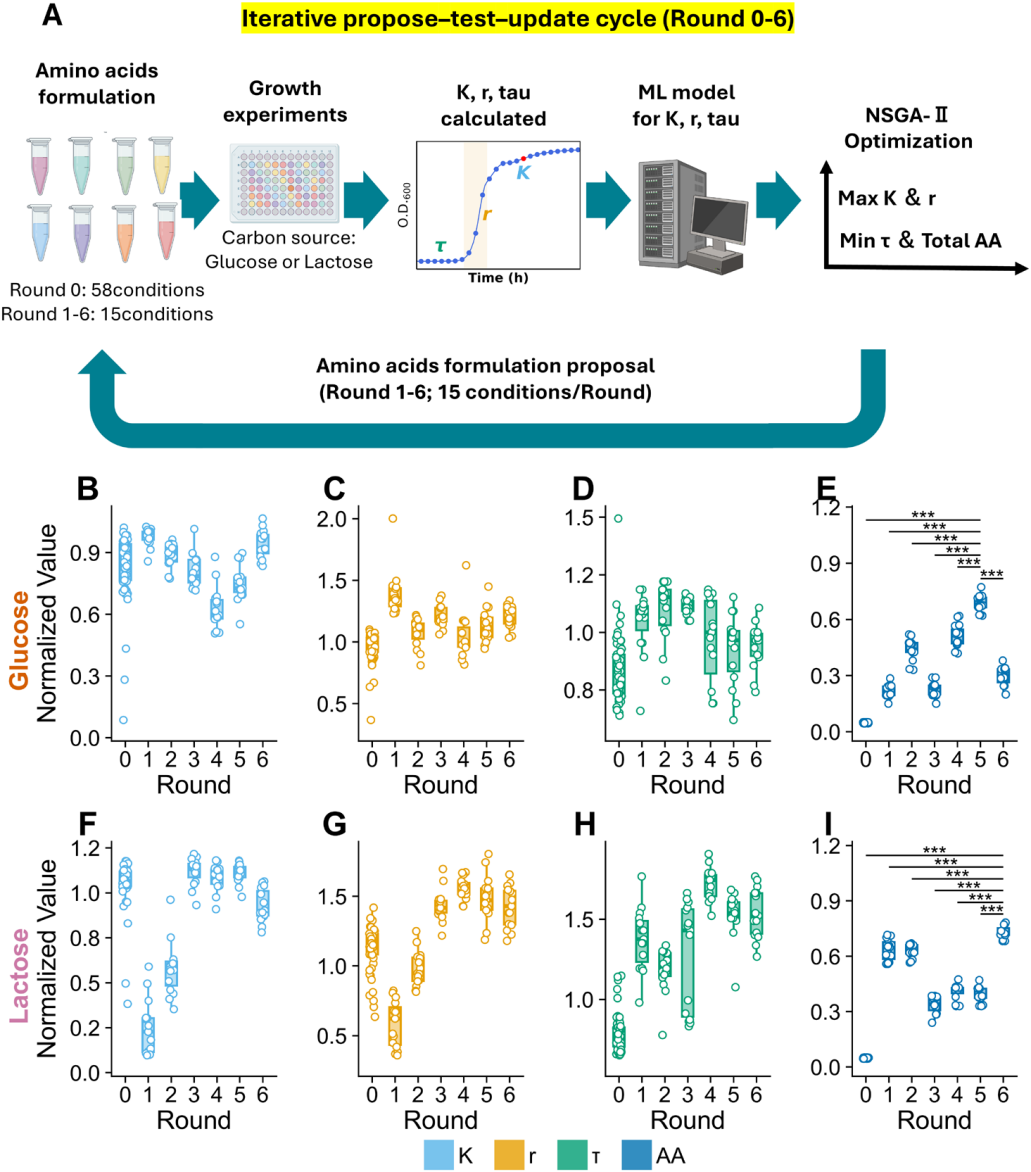
Iterative, model‐guided optimization of amino acid composition. (A) Schematic of the iterative propose–test–update cycle for model‐guided optimization of amino acid composition. Amino acid formulations were experimentally tested with glucose or lactose as the carbon source, and growth‐curve parameters—maximum cell density (K), specific growth rate (r), and lag time (τ)—were calculated. These data were used to train machine‐learning (ML) models for K, r, and τ, and NSGA‐II optimization was applied to propose new amino acid formulations to maximize K and r and minimize τ and total amino acids (Total AA). Optimization was performed by repeating this procedure six times (Rounds 1–6). As Round 0, the initial amino acid formulations consisted of 58 conditions shown in Figure S8, whereas in Rounds 1–6, 15 proposed formulations were evaluated in each round (Figure S9). (B–I) Distribution of normalized performance metrics under glucose (top) and lactose (bottom): Normalized maximum cell density (K/K_ALL; B, F), normalized specific growth rate (r/r_ALL; C, G), inverse‐normalized lag time (1/[τ/τ_ALL]; D, H), and inverse‐normalized total amino acid amount (1/[AA_total/AA_ALL]; E, I). Each point represents one medium condition. For the amino acid depletion panels (E, I), Dunnett's test was performed using the best‐performing round as the control (R5 for glucose, R6 for lactose) against all other rounds: *p < 0.05, **p < 0.01, ***p < 0.001.

Given that binary auxotrophy alone failed to explain growth performance, we next sought a quantitative framework to systematically evaluate amino acid effects on growth phenotypes (Figure 3A). To systematically quantify the influence of individual amino acids on growth phenotypes, we first generated an initial set of media conditions (Round 0; R0). For each of the 19 amino acids other than cysteine, the concentration was varied independently at three levels (4, 40, and 400 μM), producing 57 conditions, plus an ALL reference Figure S8. Under both glucose and lactose, 72‐h growth curves were obtained and used to estimate the maximum cell density (K), specific growth rate (r), and lag time (τ).

Using amino acid compositions as features and K, r, and τ as targets, we trained regression models and implemented an iterative optimization workflow. In each round, a multi‐objective genetic algorithm (NSGA‐II) proposed 15 candidate media aimed at improving growth performance while reducing total amino acid input. These candidates were experimentally tested, appended to the dataset, and used to retrain the models. This propose–test–update cycle was repeated for six rounds (R1–R6) Figure S9 A, B, Table S4. Across rounds, growth outcomes exhibited wide variation (Figure S10), indicating that amino acid composition substantially influenced both growth yield and growth kinetics. However, model prediction error (RMSE) did not consistently decrease with additional rounds (Figure S11), suggesting that the optimization explored a broad solution space rather than converging toward a single optimum.

To compare performance across rounds, we calculated four normalized metrics: K, r, τ (expressed as 1/[τ/τ_ALL]), and total amino acid input (AA; expressed as 1/[AA/AA_ALL]) (Figure 3B–I). Under glucose, R5 yielded solutions with markedly reduced AA while maintaining strong growth performance, whereas under lactose, analogous results were observed in R6.

### 3.3 Evaluation of Optimized Media Under Constrained Amino‐Acid Supply

**FIGURE 4:**
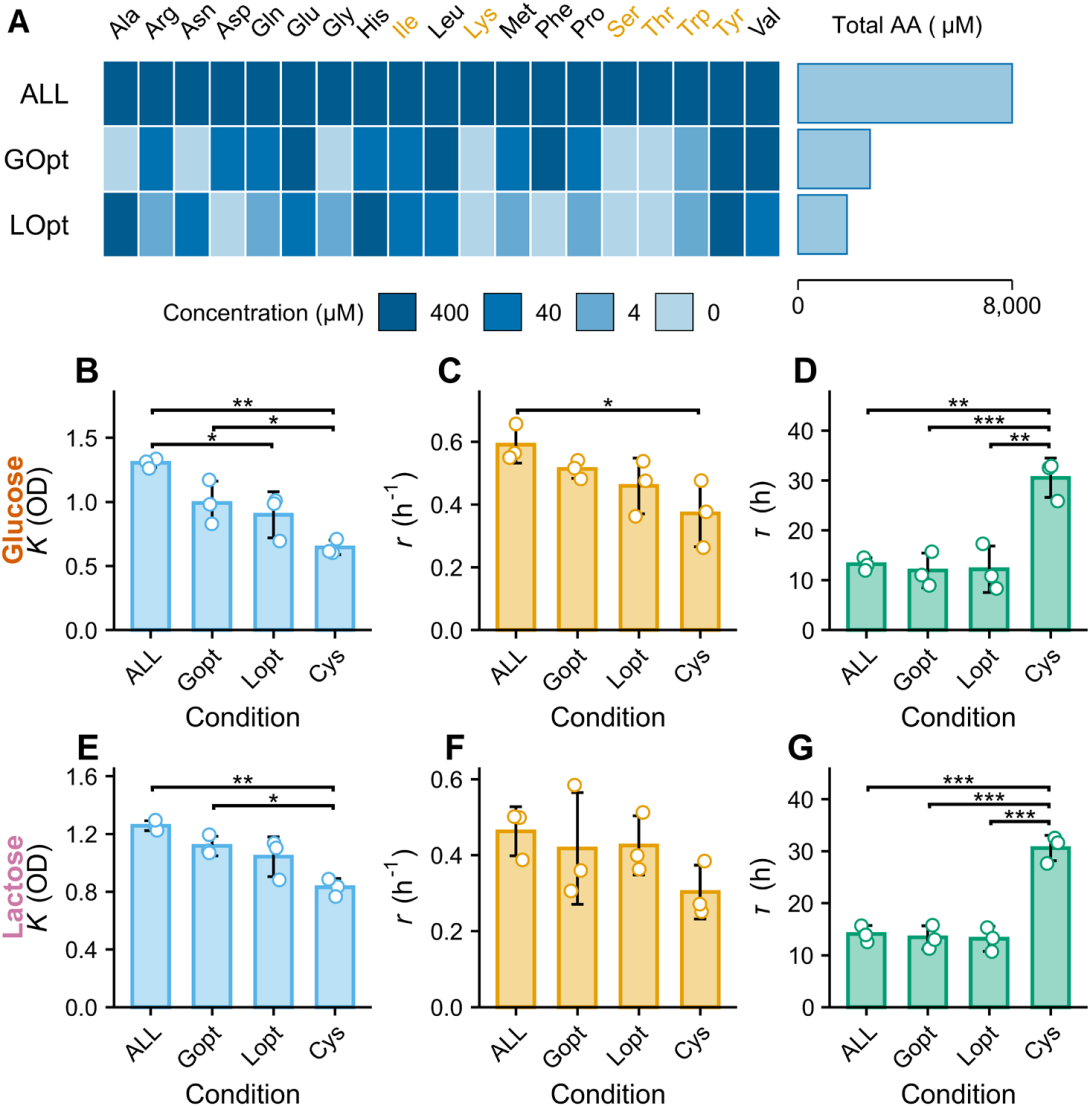
Growth performance in optimized low–amino acid media. (A) Amino acid compositions of the ALL medium and the optimized glucose (Gopt) and lactose (Lopt) media. Gopt and Lopt were selected from R5 and R6, respectively. Colour intensity indicates concentration (0/4/40/400 μM). Orange amino acid labels denote residues assigned identical concentrations in both Gopt and Lopt. Horizontal bars on the right show total amino acid content for each condition. (B–G) Growth parameters for ALL, Gopt, Lopt, and Cys under glucose (B–D) and lactose (E–G): Maximum cell density K (B, E), specific growth rate r (C, F), and lag time τ (D, G). Bars represent mean ± SD (*n* = 3 independent experiments; 4, 5 technical replicates each); points show replicates. Tukey's test: *p < 0.05, **p < 0.01, ***p < 0.001.

Based on total amino acid input, glucose R5 and lactose R6 emerged as the lowest‐AA optimization rounds. Accordingly, within each of these rounds, the medium exhibiting the highest composite performance score—defined as the sum of the four normalized metrics (K, r, τ, and total amino acids)—was selected as the glucose‐optimized (Gopt) and lactose‐optimized (Lopt) compositions, respectively (Figure S12 A, B). Their amino‐acid profiles are shown in Figure 4A, Table S6. Both formulations substantially reduced total amino acid concentration relative to the ALL medium (8000 μM total), with Gopt at 2684 μM and Lopt at 1824 μM. In Gopt, five amino acids—Glu, Leu, Phe, Tyr, and Val—were retained at the full reference concentration (400 μM), while six (Ala, Asn, Gly, Lys, Ser, and Thr) were eliminated entirely (Figure 4A, Table S6). In Lopt, only three amino acids (Ala, His, and Tyr) were retained at 400 μM, and five (Asp, Lys, Phe, Ser, and Thr) were removed. Tyrosine was the only amino acid maintained at 400 μM in both optimized media.

Under glucose, growth in Gopt did not differ significantly from ALL in K, r, or τ (Figure 4B–D). Similarly, under lactose, the Lopt medium reproduced ALL‐level performance for all three phenotypes (Figure 4E–G). In contrast, both optimized media significantly outperformed the Cys‐only medium. Gopt produced higher K and shorter τ under glucose (Figure 4B,D). While Lopt produced a significantly shorter τ under lactose (Figure 4G). These results demonstrate that growth phenotypes comparable to the ALL condition can be achieved despite a 60%–75% reduction in total amino acids.

To examine carbon‐source specificity, each optimized medium was tested with the alternative carbohydrate. Under glucose, the Lopt formulation resulted in a significantly lower K compared with ALL (Figure 4B), whereas no substantial carbon‐source dependence was observed for r or τ across conditions (Figure 4C–G). This suggests partial but not complete overlap in optimal amino acid composition between glucose and lactose growth.

### 3.4 SHAP‐Based Interpretation of Amino Acid Contributions to Growth Phenotypes

**FIGURE 5:**
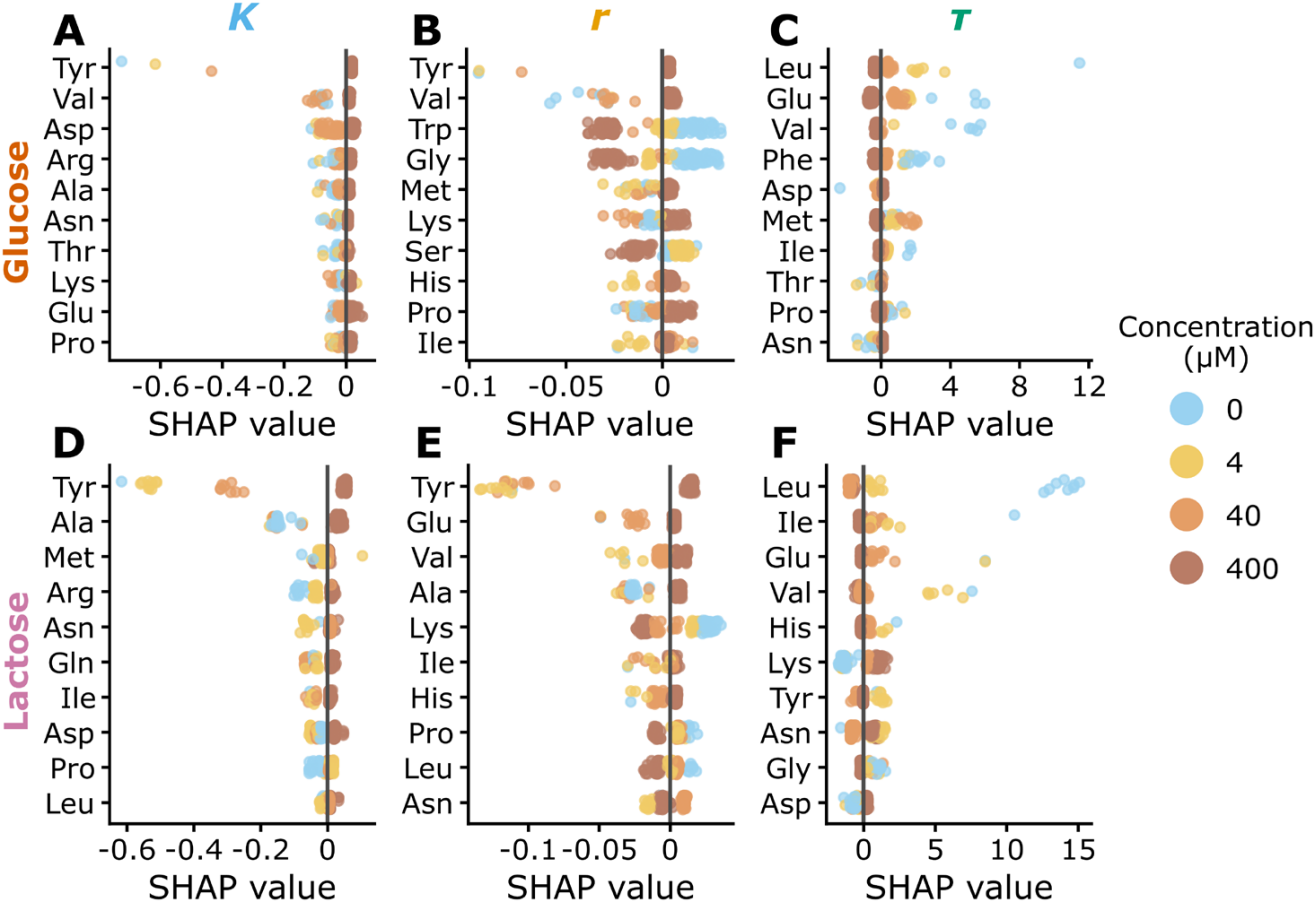
SHAP analysis of amino acid contributions to growth phenotypes. (A–F) SHAP beeswarm plots showing the top 10 amino acids contributing to model predictions of maximum cell density K (A, D), specific growth rate r (B, E), and lag time τ (C, F) under glucose (A–C) and lactose (D–F). Each dot represents one medium formulation, coloured by amino acid concentration (0/4/40/400 μM).

To identify which amino acids most strongly influenced the machine‐learning model predictions, we conducted SHAP analysis. Feature importance was defined as the mean absolute SHAP value for each amino acid at the tested concentration levels (0, 4, 40, and 400 μM) (Figure 5).

For maximum cell density (K), tyrosine (Tyr) had the highest importance under both carbon sources, indicating that variation in Tyr availability was a major determinant of final yield (Figure 5A,D). In contrast, specific growth rate (r) was influenced by a more distributed set of amino acids without a single dominant contributor (Figure 5B,E).

For lag time (τ), leucine (Leu), valine (Val), and glutamate (Glu) consistently showed the highest feature importance in both carbon sources (Figure 5C,F), suggesting that these amino acids play key roles in initiating growth under amino acid limited conditions.

Together, these analyses reveal that distinct subsets of amino acids govern different growth phenotypes: Tyr prominently influences culture yield (K), whereas Leu, Val, and Glu disproportionately shape early growth dynamics (τ), regardless of carbon source.

## 4 Discussion

While amino acid auxotrophy has traditionally been treated as a binary property, our results demonstrate that amino acids exert graded, phenotype‐specific effects on growth, which we conceptualize here as amino acid preferences. Genome‐based pathway analysis predicted a defect in cysteine (Cys) biosynthesis, and growth assays confirmed Cys as essential under our chemically defined conditions. However, supplementation with Cys alone produced markedly inferior growth—especially reduced maximum cell density (K) and prolonged lag time (τ)—compared with a complete amino acid mixture. This discrepancy highlights that binary auxotrophy predictions, based solely on pathway presence, do not capture the quantitative amino acid dependencies that influence growth kinetics and yield. Our findings align with observations in *B. longum* 105‐A, where tyrosine and isoleucine were identified as growth‐promoting factors independent of strict auxotrophy (Sakaguchi et al. 2013).

A key feature of this study was the use of iterative, model‐guided multi‐objective optimization to design amino acid compositions that sustain growth while substantially reducing total nutrient input. Under nutrient‐rich conditions, the effects of individual amino acids are largely masked, as few become limiting. By deliberately constraining total amino acid availability, we amplified phenotypic sensitivity to differences in biosynthetic cost, metabolic demand, uptake efficiency, and intracellular routing. The optimized media (Gopt and Lopt) achieved 60%–75% reductions in total amino acids while maintaining growth phenotypes comparable to the fully supplemented condition. These results support the concept that amino acid preferences—defined here as quantitative growth contributions—are more readily revealed under resource‐limited regimes.

The ecological implications of such preferences may be substantial. In the gut, amino acids serve as competitive resources, and microbial consumption can reduce luminal availability in ways that affect colonization of both commensals and pathogens (Caballero‐Flores et al. 2020, 2023). Moreover, in fluctuating environments, even modest differences in lag time or early specific growth rate can strongly impact competitive fitness, as demonstrated in *Escherichia coli* and other species (Adkar et al. 2017; Basan et al. 2020). From this perspective, amino acid preferences detected under constrained supply may indicate metabolic strategies relevant to establishment and persistence in nutrient‐variable gut ecosystems, particularly in the infant gut where bifidobacteria are abundant.

Model interpretability analysis identified distinct sets of amino acids governing different aspects of growth. Tyrosine (Tyr) was the strongest contributor to variation in K across both carbon sources, consistent with prior evidence that Tyr acts as a growth‐enhancing factor in *B. longum* (Sakaguchi et al. 2013). By contrast, lag time (τ) was most strongly influenced by glutamate (Glu), leucine (Leu), and valine (Val). Glu functions as a central node of nitrogen metabolism and participates extensively in transamination reactions; exogenous Glu may therefore accelerate metabolic adjustment and shorten lag phase (Gunka and Commichau 2012; Reitzer 2004; Walker and van der Donk 2016). Leu and Val are among the most biosynthetically costly and frequently used amino acids (Akashi and Gojobori 2002) (Figure S13), suggesting that limitation in their supply could readily delay outgrowth. Additionally, the shared biosynthetic intermediate 2‐ketoisovalerate (from Val) may partially alleviate Leu limitation, providing a mechanistic basis for the strong influence of both amino acids (Freundlich et al. 1962; Koon et al. 2004; Li et al. 2017). Similar relationships reported in *E. coli* (Varik et al. 2016) suggest that these amino acid dependent effects on early growth may reflect general bacterial physiology.

The amino acid preferences identified in this study share common features with those reported in other bifidobacteria. In *B. breve* , aromatic lactic acid production produced from aromatic amino acids provides a direct fitness advantage that markedly promotes growth (Shiver et al. 2025). Our observation that tyrosine is the strongest determinant of maximum cell density is consistent with the findings of Shiver et al., and suggests that in *B. longum* , tyrosine contributes to growth not only as a proteinogenic amino acid but also, at least in part, through its role in redox cofactor regeneration. Although the relative importance of individual aromatic amino acids is likely to vary depending on species‐ or strain‐specific enzyme properties and cultivation conditions, the reliance on aromatic amino acid metabolism as a core component of growth strategies under nutrient limitation may represent a conserved feature across bifidobacteria. Moreover, because the production of aromatic lactic acids and related signalling molecules—including indole‐3‐acetate and indole‐3‐carboxaldehyde—scales with exogenous aromatic amino acid supply (Zhang et al. 2025; Plenge et al. 2025), the amino acid preferences quantified here may influence not only growth but also the profile of metabolites relevant to host physiology.

Comparison of optimized amino acid compositions for glucose versus lactose revealed both shared and flexible requirements. Tyr, Glu, Leu, and Val were consistently retained in both optimized media, suggesting a conserved core set of amino acids that robustly supports growth across varying carbon sources. At the same time, other components appeared interchangeable, and switching carbon sources did not broadly impair growth. This pattern implies that amino acid preference landscapes may contain multiple near‐equivalent optima, enabling gut commensals to maintain growth across diverse nutrient environments. Extending this analysis to additional carbon sources or to carbon‐limited conditions could further delineate how carbon metabolism constrains or reshapes amino acid requirements.

Methodologically, our approach integrates high‐throughput phenotyping, machine‐learning regression, and multi‐objective genetic optimization into a unified iterative framework. This strategy is broadly applicable to optimizing nutrient formulations, characterizing metabolic dependencies, and designing microbial consortia where nutrient allocation influences stability and function. Potential applications include improving media for probiotic manufacturing, designing defined media for disease‐relevant in vitro models, and tuning nutrient profiles in co‐culture systems to modulate competitive dynamics. By jointly optimizing growth phenotypes and nutrient input, our framework offers a practical route to simplifying media while maintaining target performance.

Several limitations should be noted. First, our analyses were performed in monoculture using a single strain in chemically defined media, which does not capture the complexity of gut environments involving peptides, microbial cross‐feeding, and host‐derived nutrients. Second, amino acids were tested at discrete concentration levels, and we did not directly measure transport rates, intracellular pools, or flux distributions; mechanistic interpretation therefore remains inferential. Future studies combining gene perturbation, metabolomics, flux analysis, and co‐culture experiments will be essential to establish causal pathways underlying the inferred preferences. Finally, extending the analysis to additional strains and species will enable assessment of how conserved versus strain‐specific amino acid preferences are within *Bifidobacterium* and across gut commensals.

In summary, this study moves beyond binary assessments of auxotrophy and provides quantitative data on how individual amino acids modulate growth phenotypes under defined conditions. Through iterative, model‐guided optimization, we identified amino acid compositions that dramatically reduce nutrient input while preserving growth. These findings provide new insight into amino acid utilization strategies in *B. longum* and establish a generalizable framework for data‐driven medium optimization in microbiology and biotechnology.

## Author Contributions

**Shin Yoshimoto:** conceptualization, project administration, supervision, visualization, writing – original draft, writing – review and editing. **Hiroki Kaneko:** conceptualization, investigation, writing – original draft, writing – review and editing, validation, methodology, software, data curation, formal analysis, visualization, resources. **Bei ‐ Wen Ying:** conceptualization, methodology, project administration, supervision, visualization, writing – review and editing. **Toshitaka Odamaki:** conceptualization, funding acquisition, project administration, supervision, visualization, writing – review and editing. **Kana Kadowaki:** investigation, methodology, resources.

## Funding

This work was supported by Morinaga Milk Industry.

## Conflicts of Interest

Hiroki Kaneko, Kana Kadowaki, Shin Yoshimoto, and Toshitaka Odamaki are employees of Morinaga Milk Industry Co. Ltd which has several probiotic products marketed worldwide.

## Supporting information


**Figure S1:** Amino acid biosynthetic pathways: pyruvate family.
**Figure S2:** Amino acid biosynthetic pathways: oxaloacetate family (part 1).
**Figure S3:** Amino acid biosynthetic pathways: oxaloacetate family (part 2).
**Figure S4:** Amino acid biosynthetic pathways: 2‐oxoglutarate family.
**Figure S5:** Amino acid biosynthetic pathways: 3‐phosphoglycerate family.
**Figure S6:** Amino acid biosynthetic pathways: aromatic family.
**Figure S7:** Amino acid biosynthetic pathway: histidine.
**Figure S8:** (Related to Figure 3) Amino‐acid composition of the initial media in Round 0 (R0) (58 conditions).
**Figure S9:** Changes in amino‐acid compositions proposed by NSGA‐II across rounds R1–R6.
**Figure S10:** Distribution of growth parameters across all tested media conditions.
**Figure S11:** Model prediction error across optimization rounds.
**Figure S12:** Composite performance scores across optimization rounds.
**Figure S13:** Amino acid usage and biosynthetic energy demand in the B. longum JCM 1217T proteome.

**Table S1:** Integrated KEGG Ortholog (KO) annotations for *B. longum* JCM 1217^T^ derived from KOfamScan and eggNOG‐mapper.
**Table S2:** Composition of the 2× concentrated chemically defined medium (2 × CDM) used in this study.
**Table S3:** Completeness of amino‐acid biosynthetic pathways in B. longum JCM 1217T.
**Table S4:** Amino‐acid compositions and growth parameters for 296 medium conditions across six optimization rounds.
**Table S5:** Machine learning model performance across active learning rounds.
**Table S6:** Amino‐acid compositions and growth phenotypes of optimized media (Gopt and Lopt).

## Data Availability

The data that supports the findings of this study are available in the supporting information of this article.
