# Supplementary material for: Machine Learning Reveals Quantitative Amino Acid Preferences in Bifidobacterium longum Growth
Source: Microb Biotechnol. 2026 May 5;19(5):e70367. doi: 10.1111/1751-7915.70367 (PMC13144548; doi:10.1111/1751-7915.70367)
Supplement: Supplementary file 1 — Figure S1: Amino acid biosynthetic pathways: pyruvate family. Figure S2: Amino acid biosynthetic pathways: oxaloacetate family (part 1). Figure S3: Amino acid biosynthetic pathways: oxaloacetate family (part 2). Figure S4: Amino acid biosynthetic pathways: 2‐oxoglutarate family. Figure S5: Amino acid biosynthetic pathways: 3‐phosphoglycerate family. Figure S6: Amino acid biosynthetic pathways: aromatic family. Figure S7: Amino acid biosynthetic pathway: histidine. Figure S8: (Related to Figure 3) Amino‐acid composition of the initial media in Round 0 (R0) (58 conditions). Figure S9: Changes in amino‐acid compositions proposed by NSGA‐II across rounds R1–R6. Figure S10: Distribution of growth parameters across all tested media conditions. Figure S11: Model prediction error across optimization rounds. Figure S12: Composite performance scores across optimization rounds. Figure S13: Amino acid usage and biosynthetic energy demand in the B. longum JCM 1217T proteome. [file MBT2-19-e70367-s001.docx]

**Supporting Information**

**TableS1. Integrated KEGG Ortholog (KO) annotations for *B. longum* JCM 1217^T^ derived from KOfamScan and eggNOG-mapper**

**TableS2. Composition of the 2x concentrated chemically defined medium (2x CDM) used in this study**

**TableS3. Completeness of amino-acid biosynthetic pathways in *B. longum* JCM 1217^T^**

**TableS4. Amino-acid compositions and growth parameters for 296 medium conditions across six optimization rounds**

**Table S5. Machine learning model performance across active learning rounds.**

**TableS6. Amino-acid compositions and growth phenotypes of optimized media (Gopt and Lopt)**

**Figure S1. Amino acid biosynthetic pathways: pyruvate family**

**Figure S2. Amino acid biosynthetic pathways: oxaloacetate family (part 1)**

**Figure S3. Amino acid biosynthetic pathways: oxaloacetate family (part 2)**

**Figure S4. Amino acid biosynthetic pathways: 2-oxoglutarate family**

**Figure S5. Amino acid biosynthetic pathways: 3-phosphoglycerate family**

**Figure S6. Amino acid biosynthetic pathways: aromatic family**

**Figure S7. Amino acid biosynthetic pathway: histidine**

**Figure S8. (Related to Fig. 3) Amino-acid composition of the initial media in Round 0 (R0) (58 conditions)**

**Figure S9. Changes in amino-acid compositions proposed by NSGA-II across rounds R1–R6**

**Figure S10. Distribution of growth parameters across all tested media conditions**

**Figure S11. Model prediction error across optimization rounds**

**Figure S12. Composite performance scores across optimization rounds**

**Figure S13. Amino acid usage and biosynthetic energy demand in the *B. longum* JCM 1217^T^ proteome**

**``**


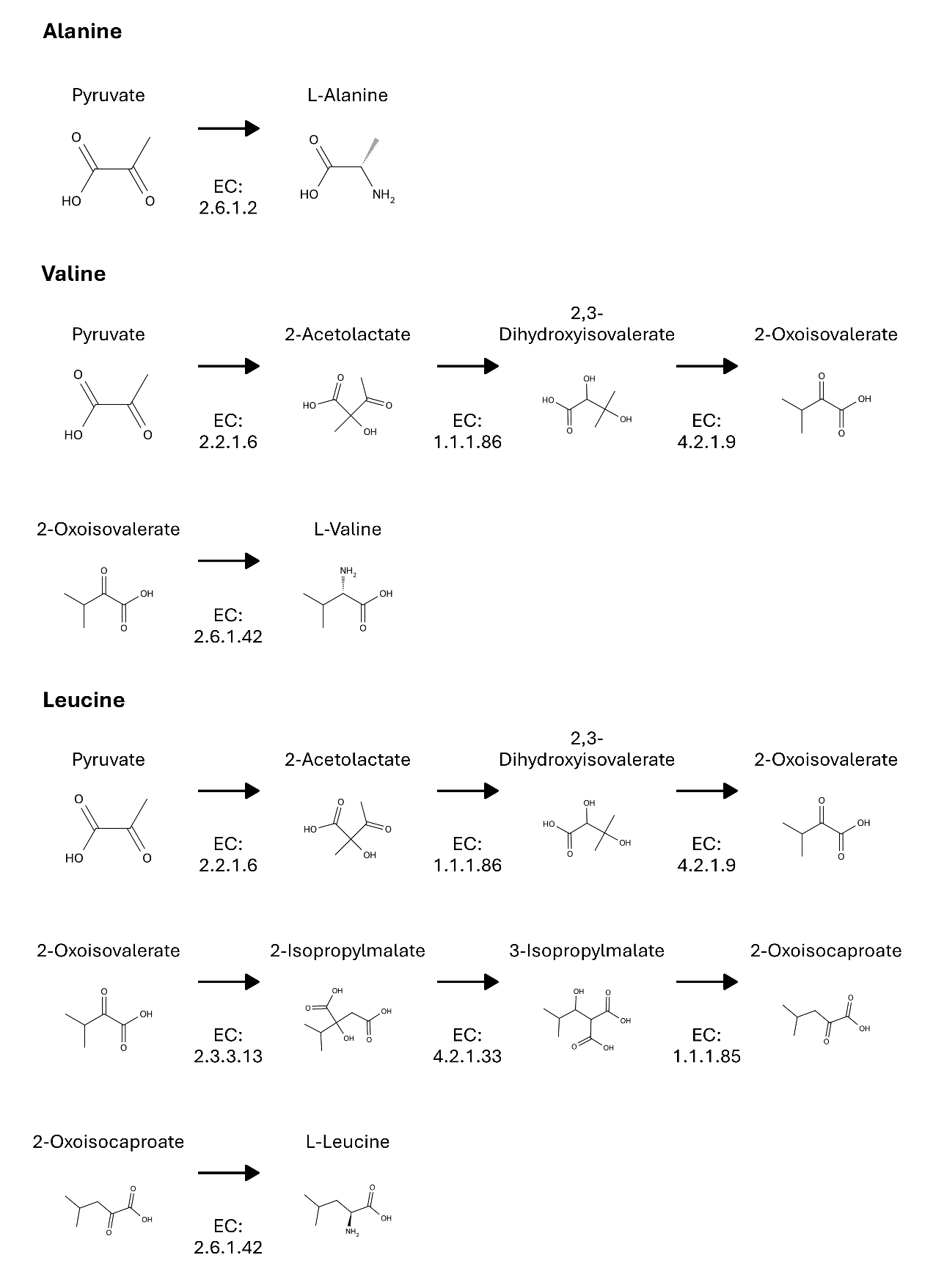
**Figure S1. Amino acid biosynthetic pathways: pyruvate family.**

Pathway diagrams showing de novo biosynthesis of alanine, valine, and leucine. Common note (for Figures S1–S7): Pathways were reconstructed and annotated based on KEGG Ortholog (KO) assignments.


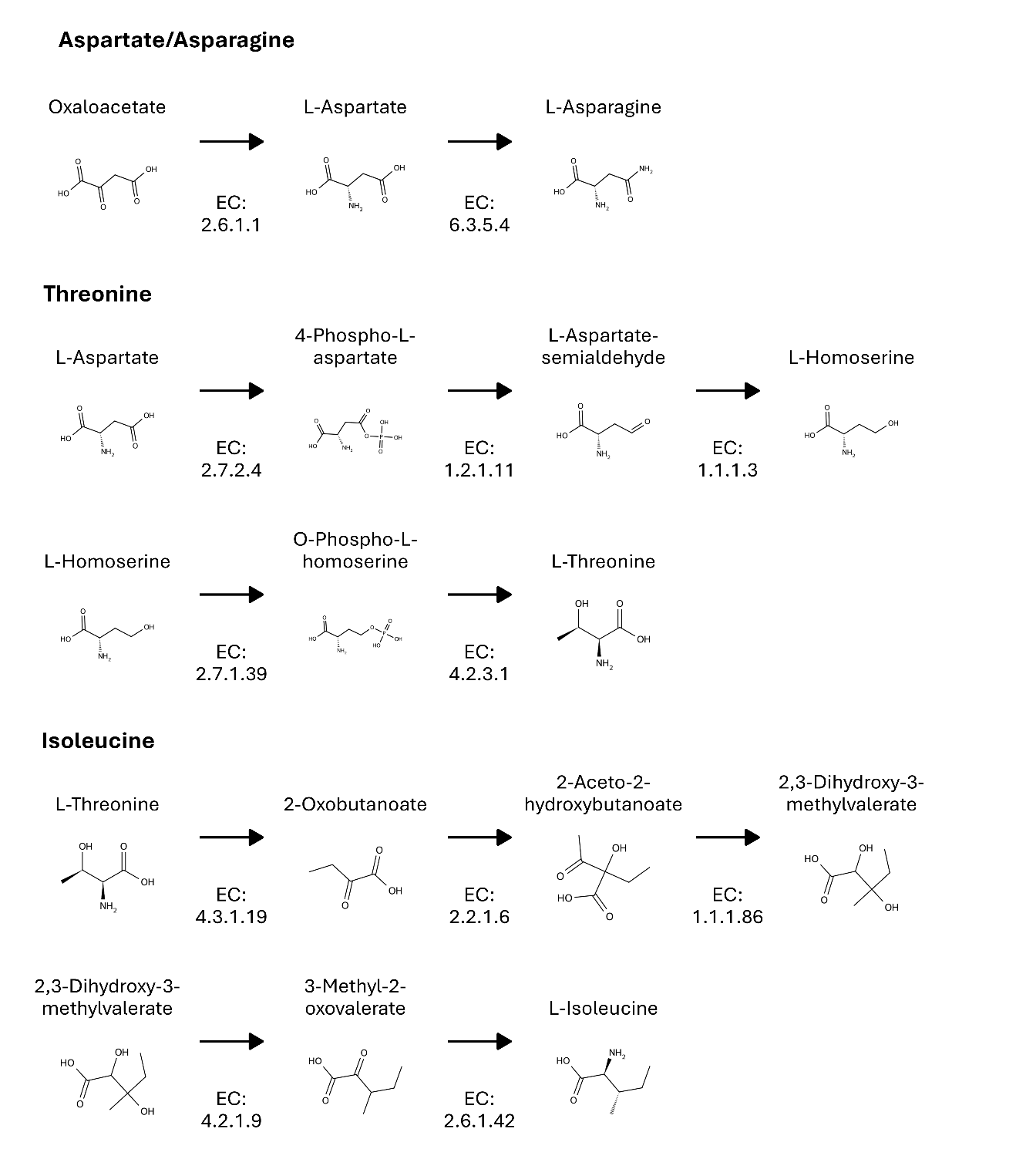


**Figure S2. Amino acid biosynthetic pathways: oxaloacetate family (part 1).**

Pathway diagrams showing de novo biosynthesis of aspartate/asparagine, threonine, and isoleucine.


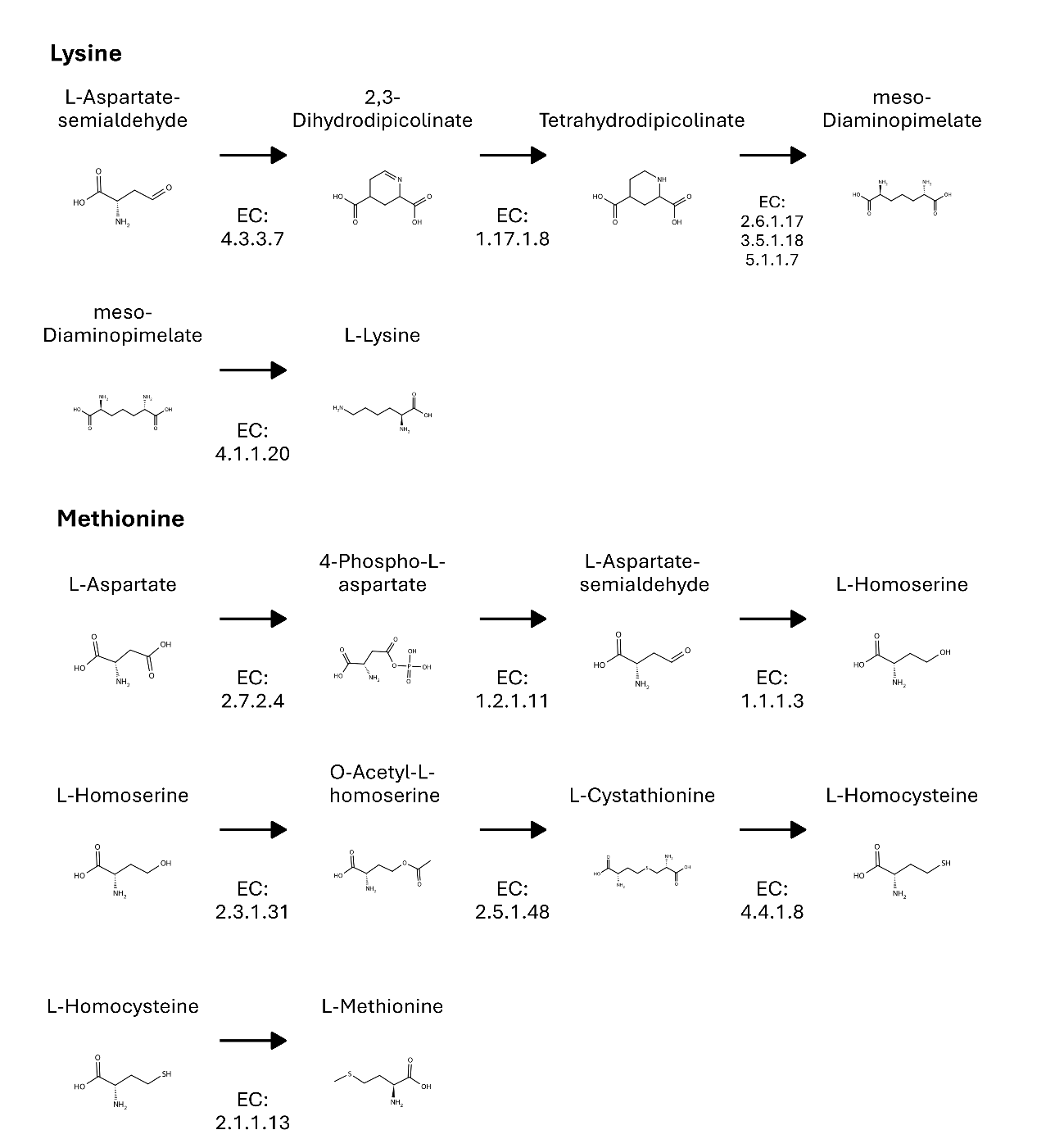


**Figure S3. Amino acid biosynthetic pathways: oxaloacetate family (part 2).**

Pathway diagrams showing de novo biosynthesis of lysine and methionine.


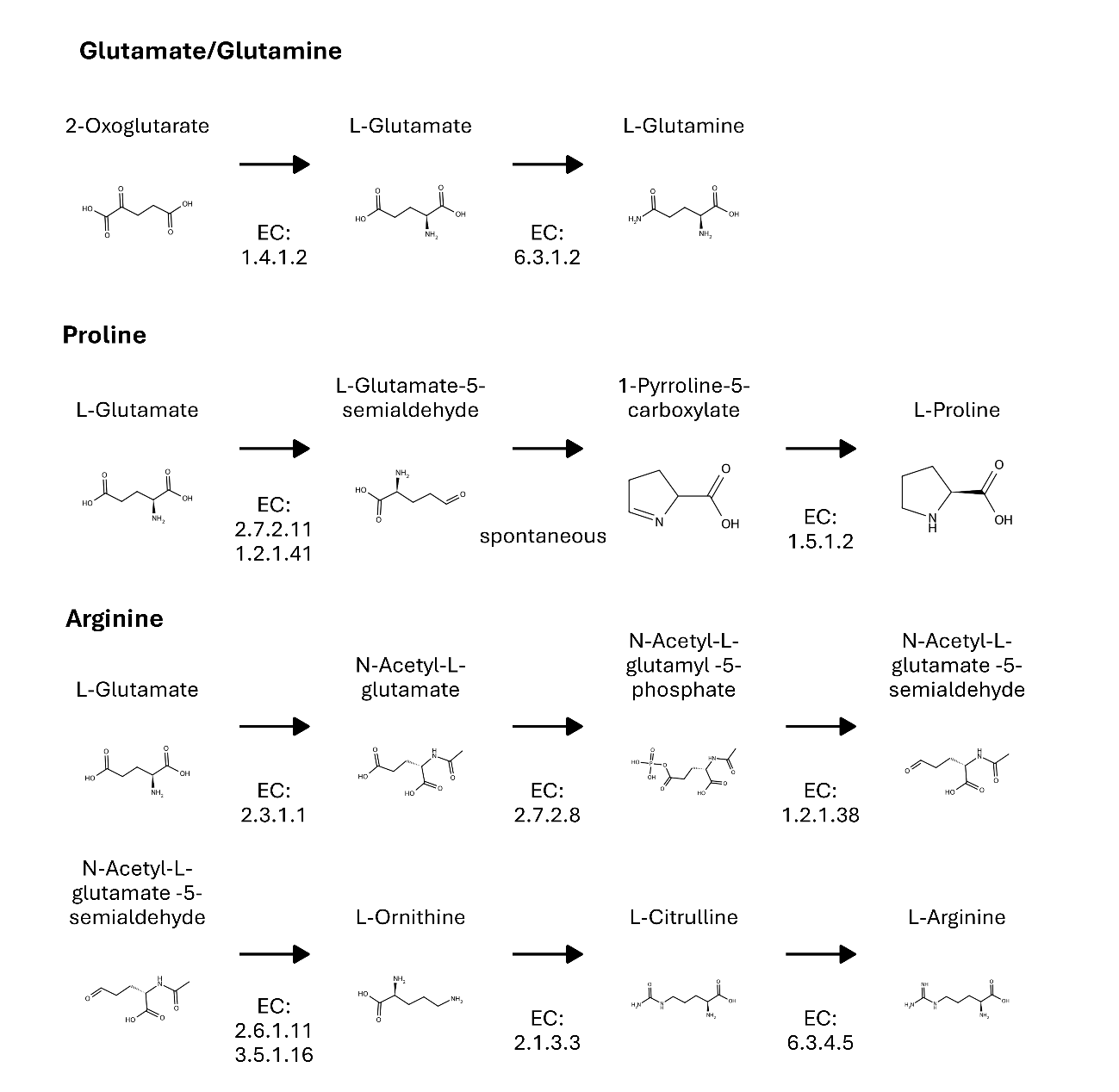


**Figure S4. Amino acid biosynthetic pathways: 2-oxoglutarate family.**

Pathway diagrams showing de novo biosynthesis of glutamate/glutamine, proline, and arginine.

**
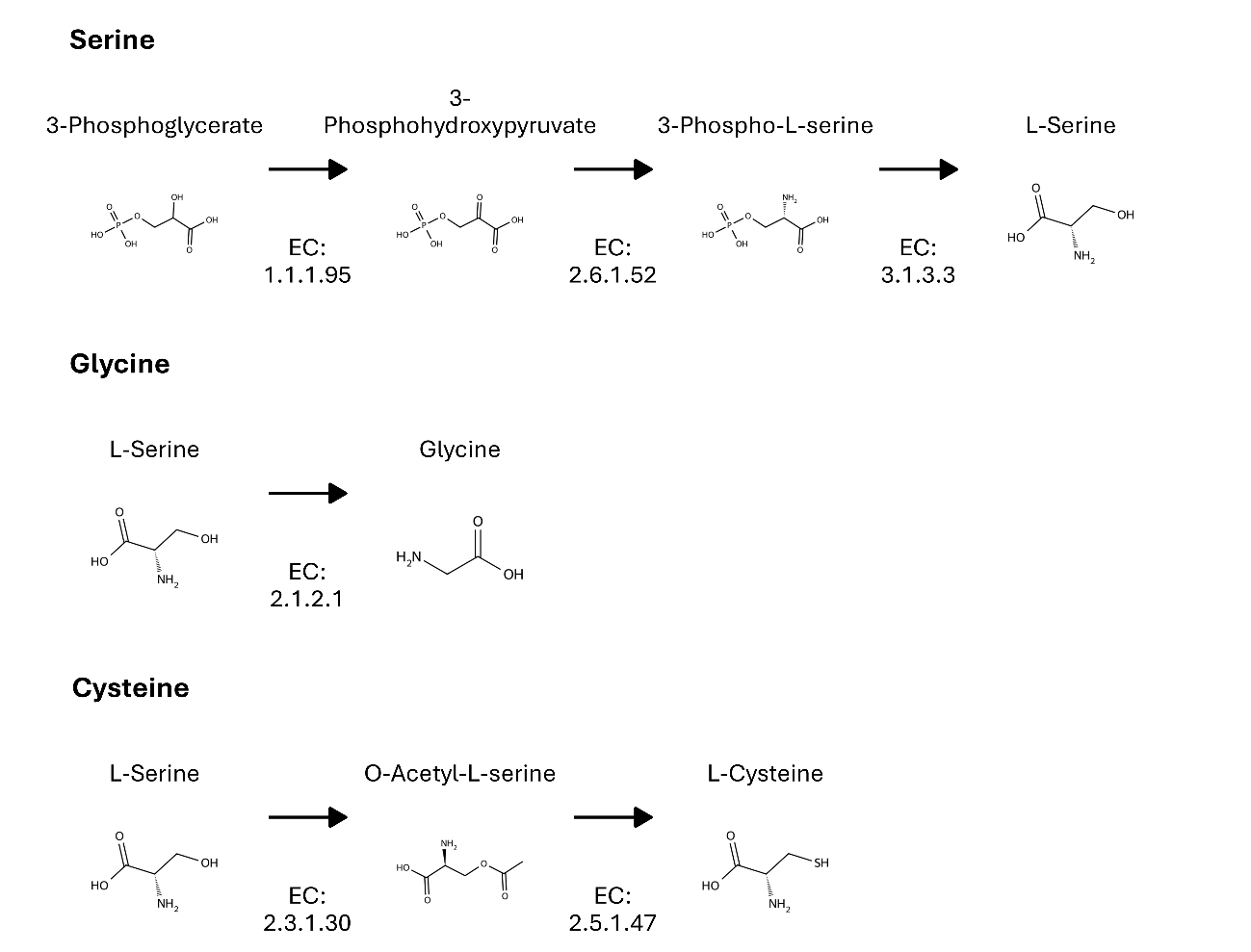
**

**Figure S5. Amino acid biosynthetic pathways: 3-phosphoglycerate family.**

Pathway diagrams showing de novo biosynthesis of serine, glycine, and cysteine.


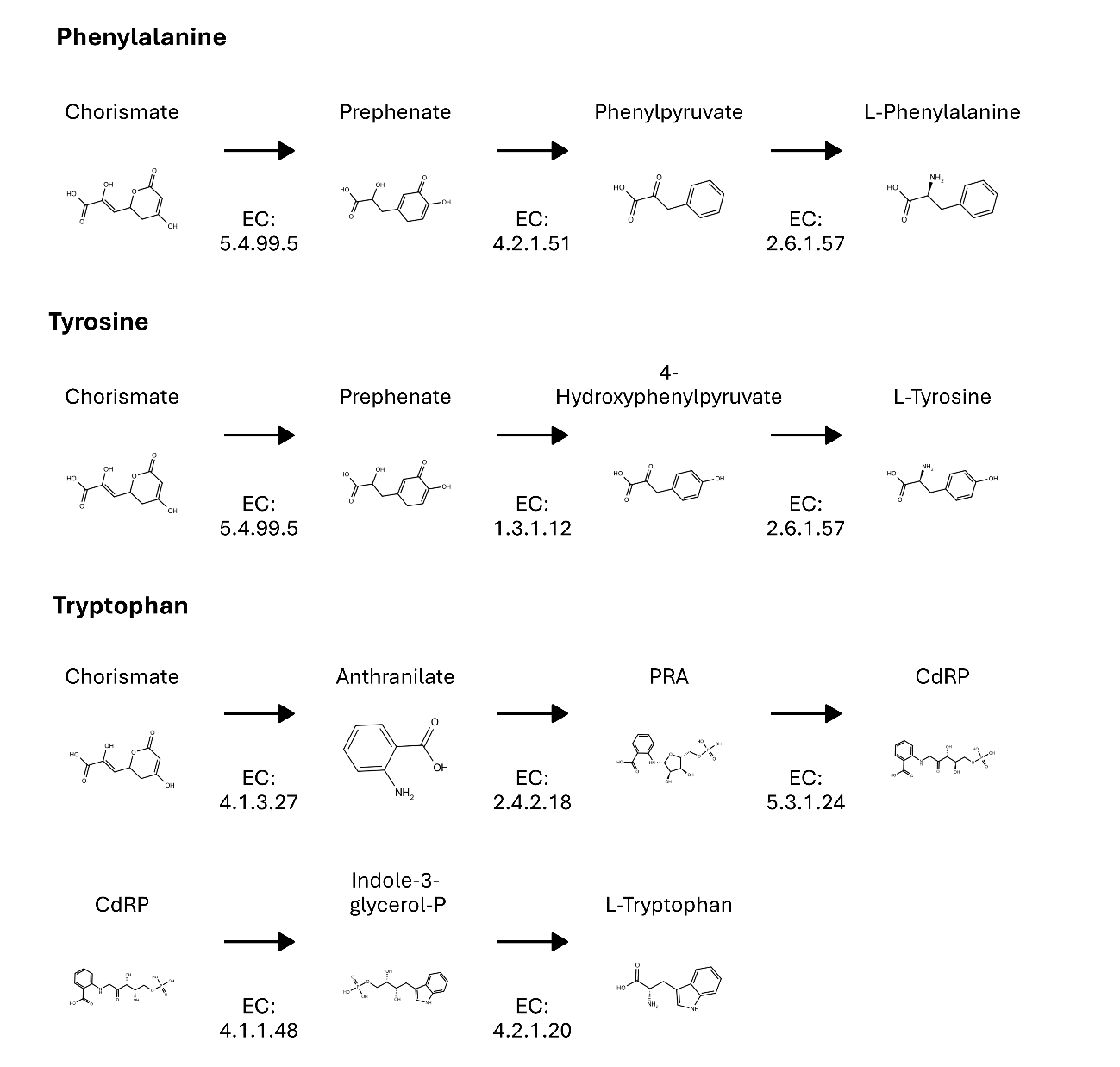


**Figure S6. Amino acid biosynthetic pathways: aromatic family.**

Pathway diagrams showing de novo biosynthesis of phenylalanine, tyrosine, and tryptophan.


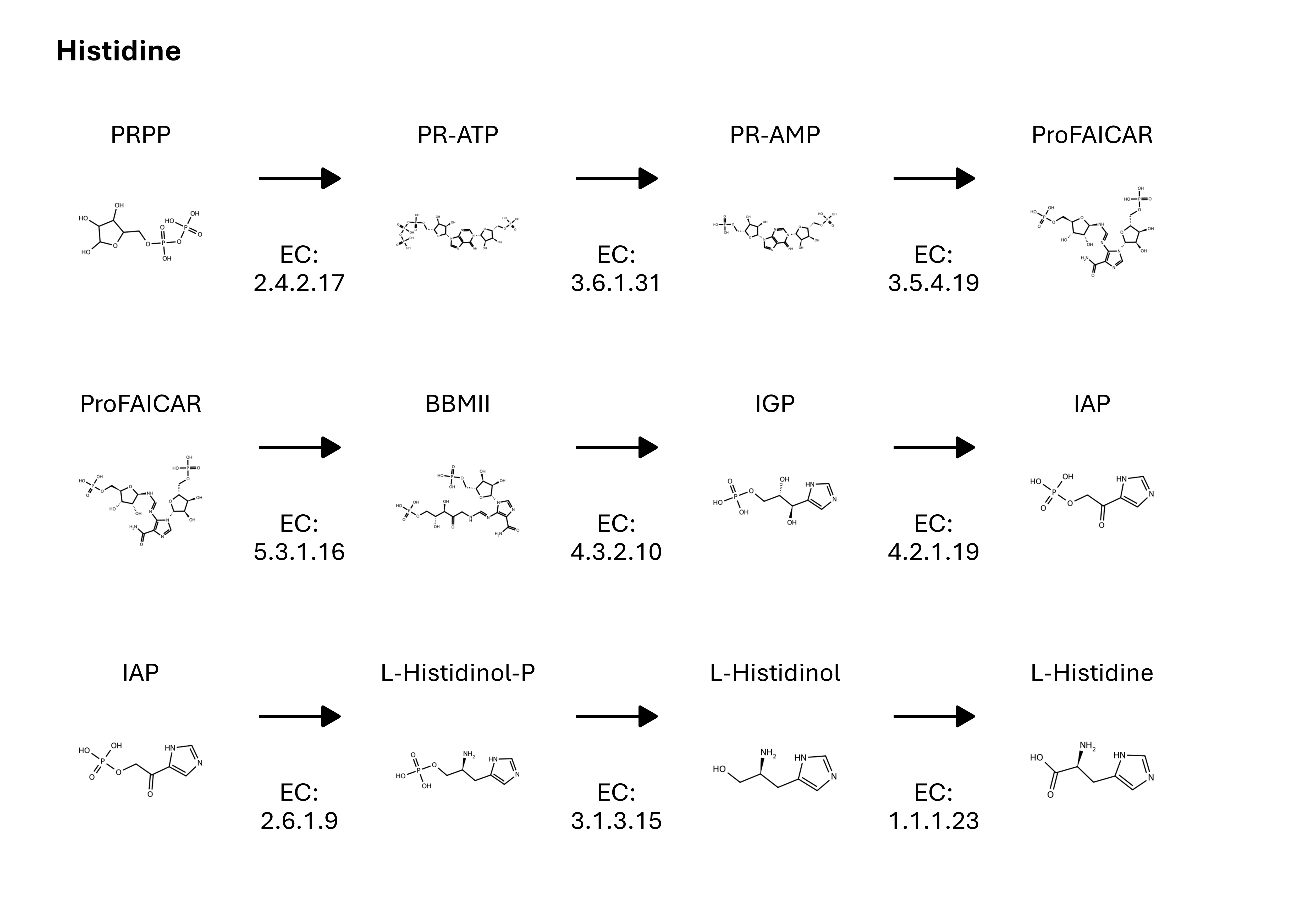


**Figure S7. Amino acid biosynthetic pathway: histidine.**

Pathway diagram showing de novo histidine biosynthesis.


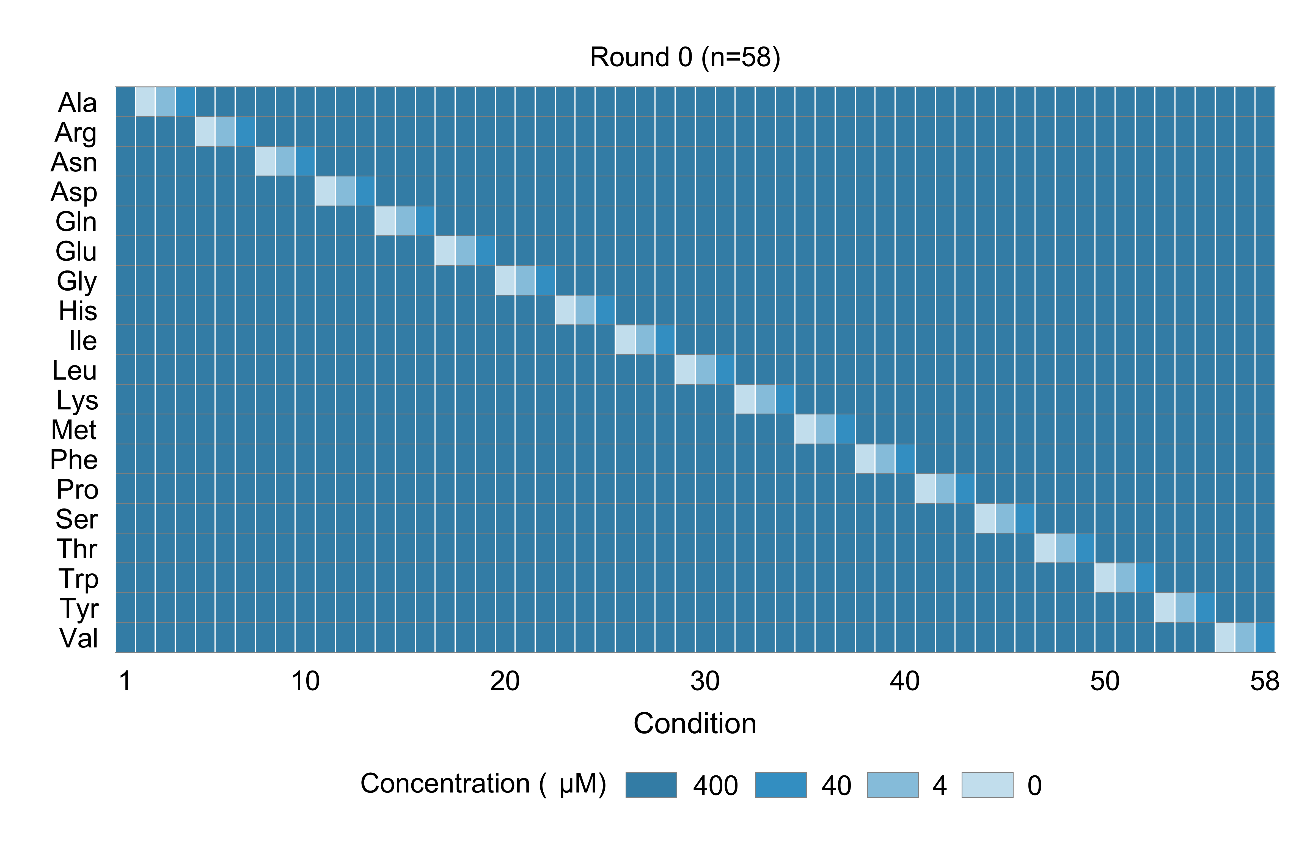


**Figure S8. (Related to Fig.3）Amino‑acid composition of the initial media in Round 0 (R0) (58 conditions).**

In R0, among the 19 amino acids excluding cysteine, the concentration of only one amino acid was varied per condition (4, 40, or 400 µM), while the remaining amino acids were all fixed at 400 µM. This design yielded a total of 58 conditions, enabling evaluation of the individual effects of each amino acid.


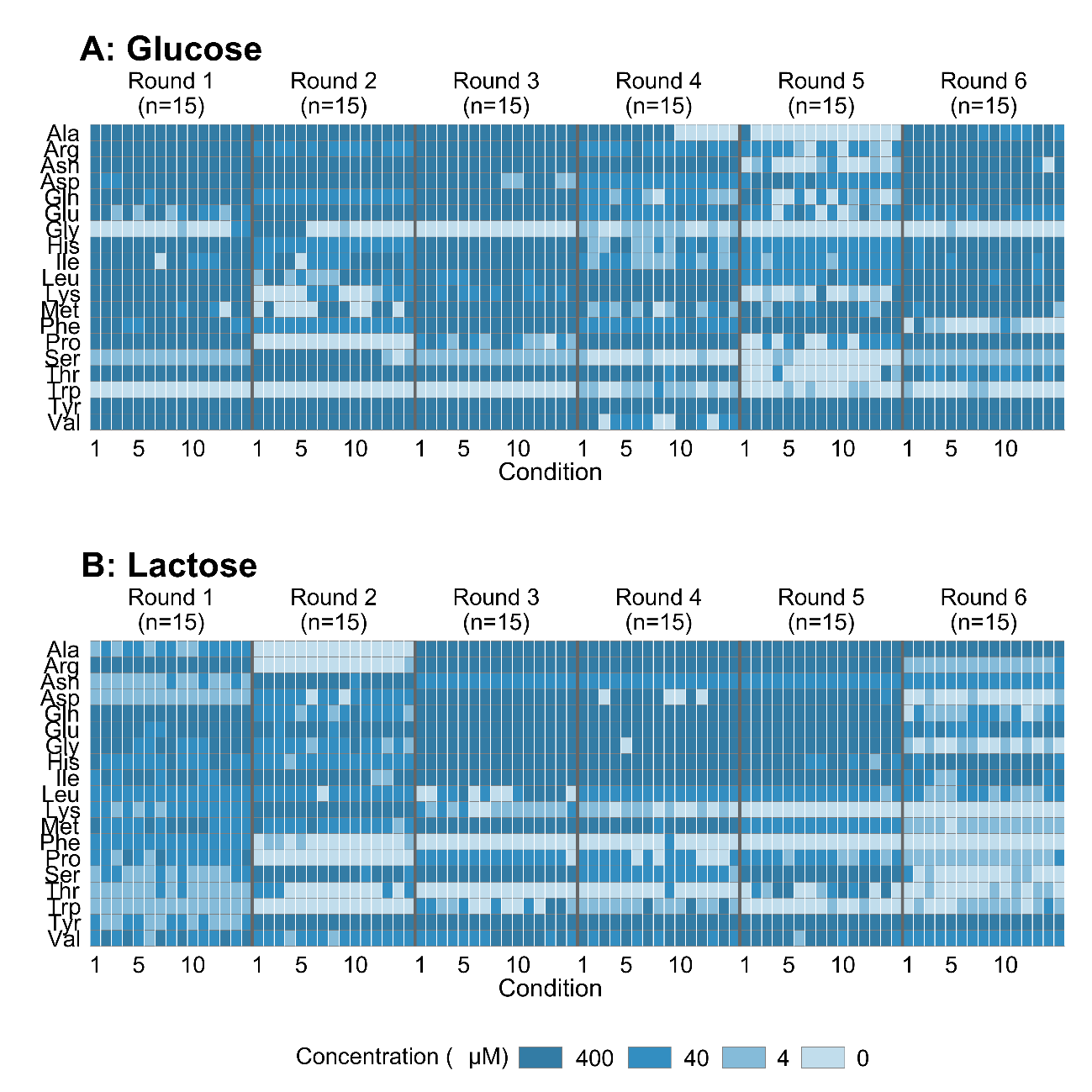


**Figure S9. Changes in amino acid compositions proposed by NSGA‑II across rounds R1–R6.**

Schematic representation of the amino‑acid compositions proposed by NSGA‑II in Rounds R1–R6 under glucose (A) and lactose (B) conditions. Colours indicate the concentration of each amino acid (0/4/40/400 µM).


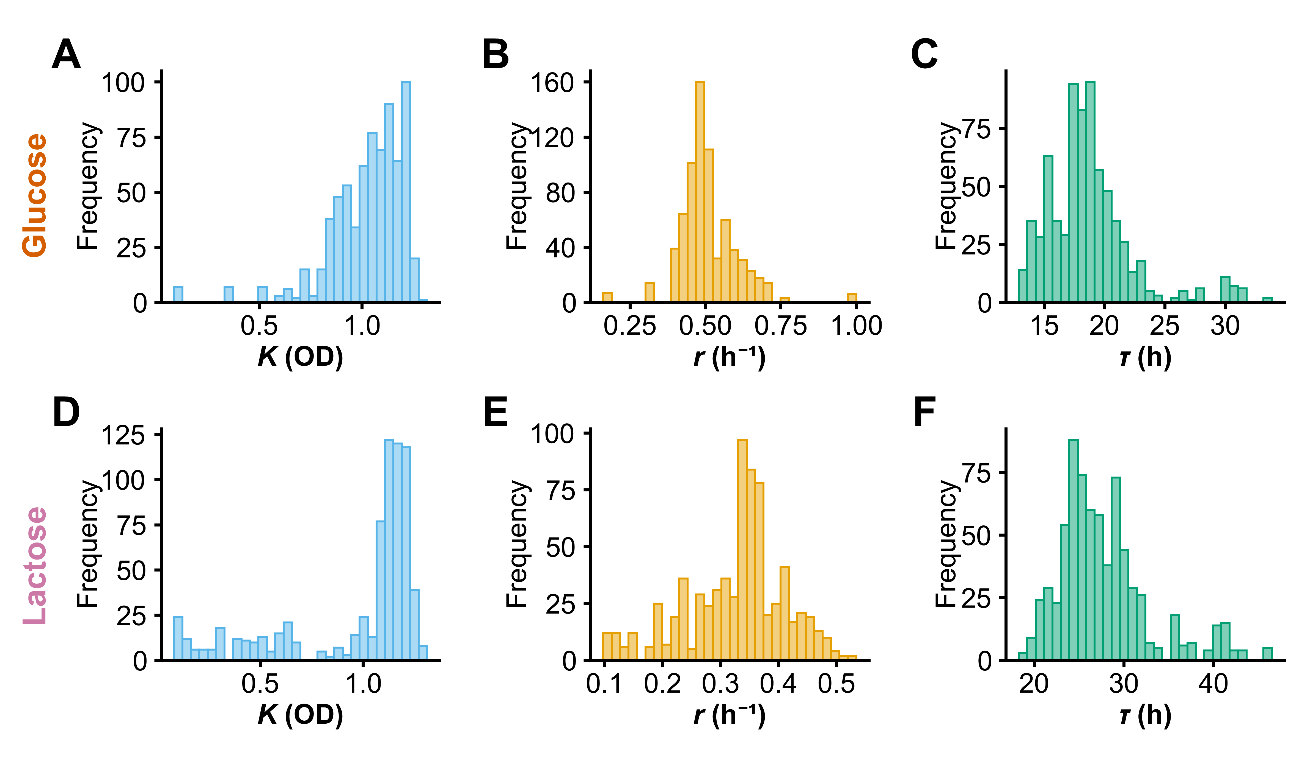
**Figure S10. Distribution of growth parameters across all tested media conditions**

(A–F) Histograms of maximum cell density K (A, D), specific growth rate r (B, E), and lag time τ (C, F) across all 296 media conditions tested during iterative optimization under glucose (A, B, C) and lactose (D, E, F).


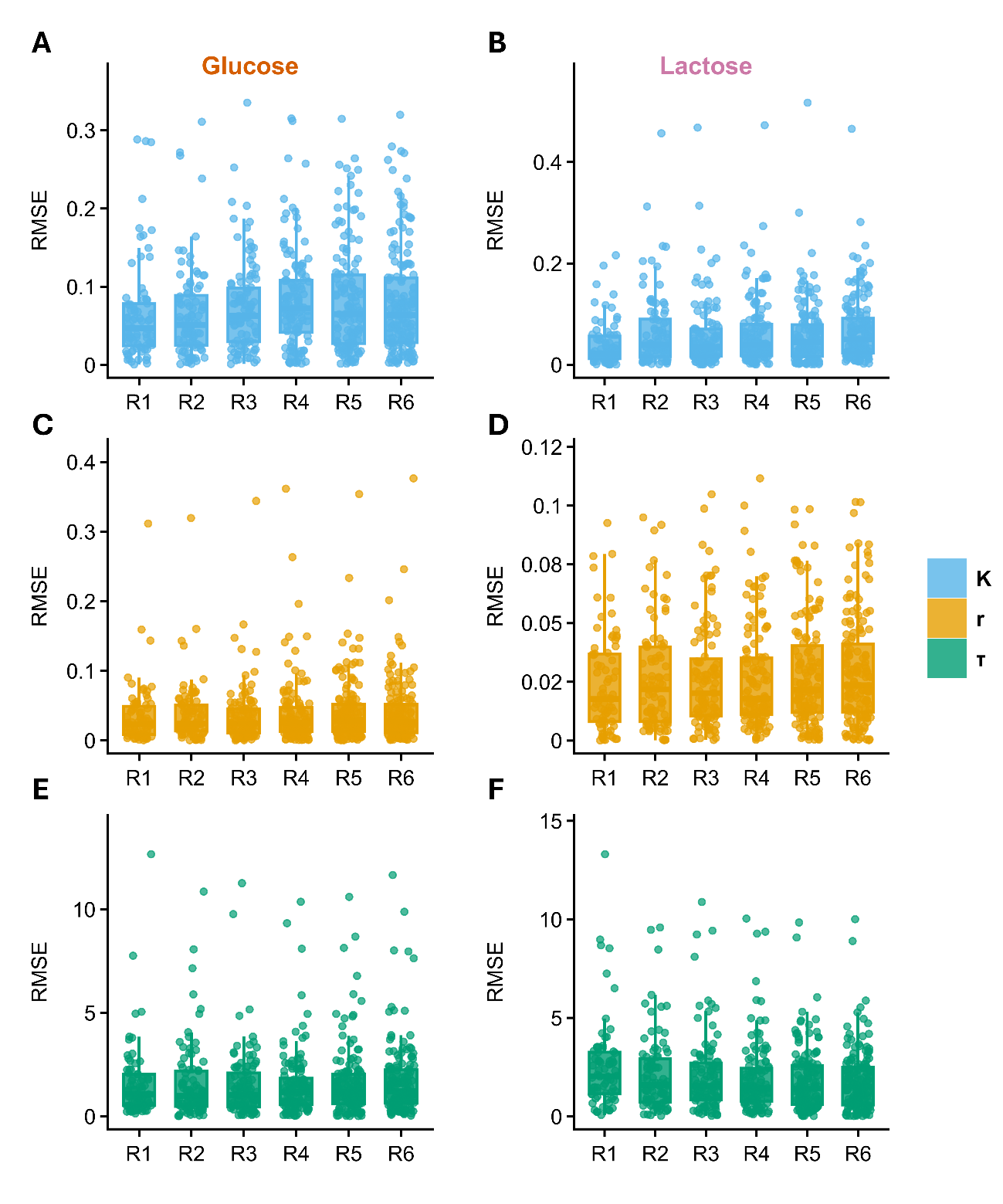


**Figure S11. Model prediction error across optimization rounds.**

Box plots showing root-mean-square error (RMSE) of machine-learning predictions for K, r, and τ across optimization rounds R1–R6 under glucose and lactose. RMSE was estimated using bootstrap leave-one-out cross-validation (200 iterations); each point represents one bootstrap resample.


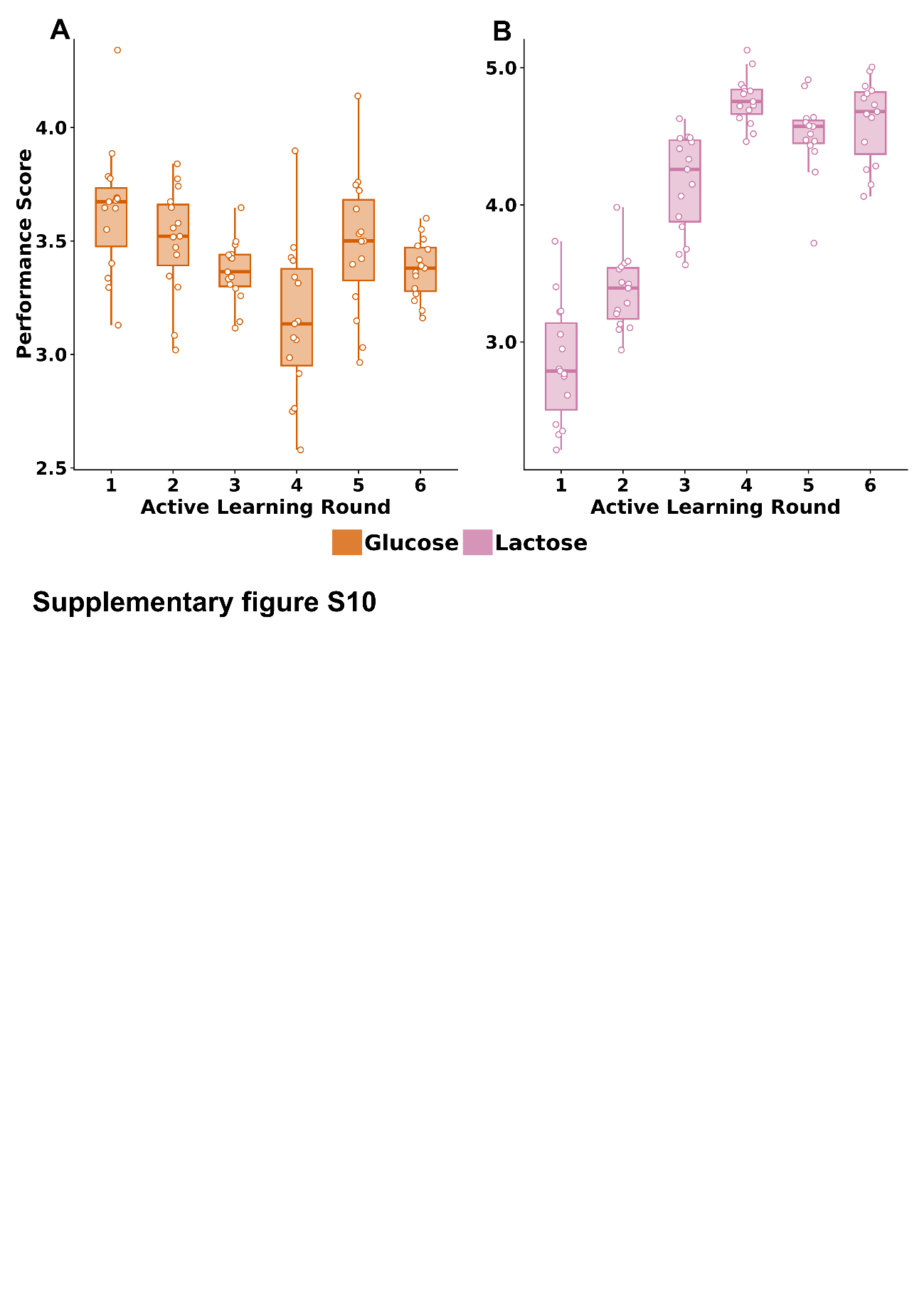


**Figure S12. Composite performance scores across optimization rounds.**

(A, B) Box plots of composite performance scores for rounds R1–R6 under glucose (A) and lactose (B) Scores were calculated as K/K_ALL + r/r_ALL + 1/(τ/τ_ALL) + 1/(AA_total/AA_ALL). Top-scoring conditions from glucose R5 and lactose R6 were selected as Gopt and Lopt, respectively.


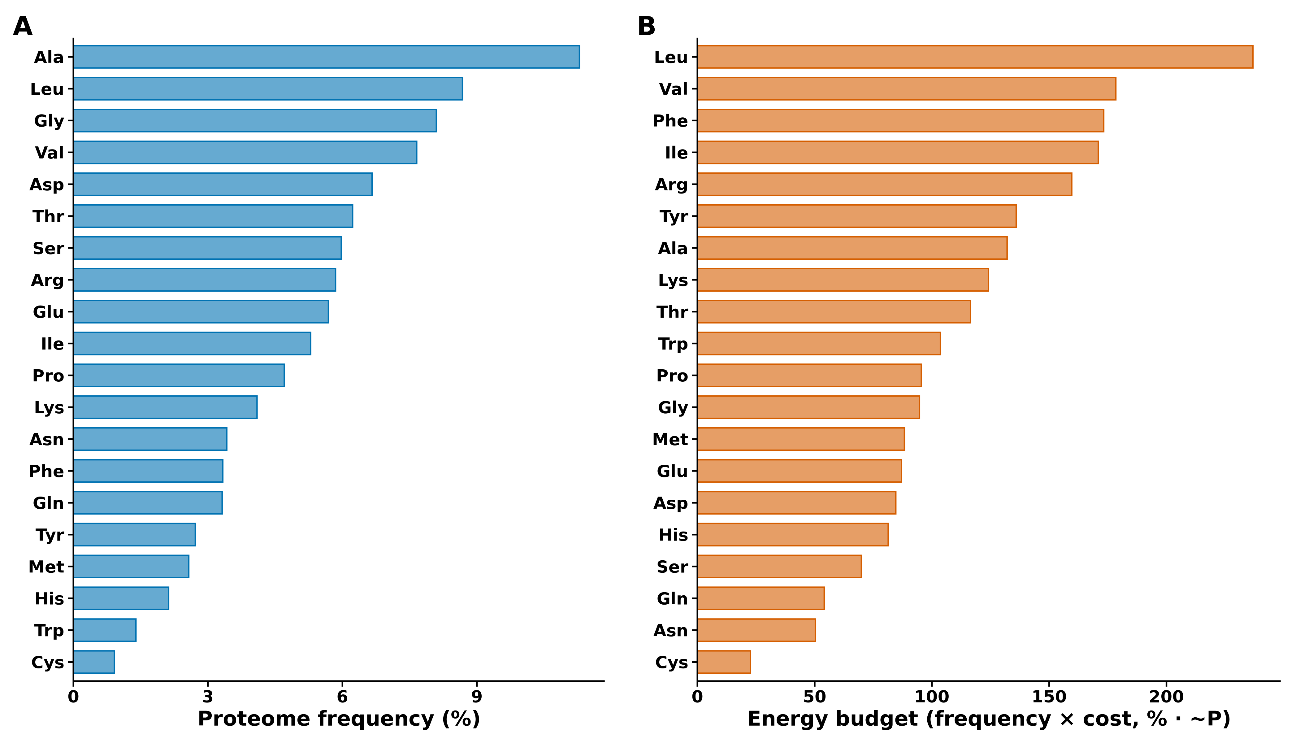


**Figure S13. Amino acid usage and biosynthetic energy demand in the *B. longum* JCM 1217^T^ proteome.**

(A) Amino-acid frequencies (%) across 1,951 predicted proteins (690,818 residues), ranked in descending order. (B) Estimated biosynthetic energy demand per amino acid, calculated as frequency (%) x de novo biosynthetic cost ( ~P equivalents) based on Akashi and Gojobori (2002), and ranked by resulting energy-demand index.
